# Super-resolution fluorescence-assisted diffraction computational tomography reveals the three-dimensional landscape of the cellular organelle interactome

**DOI:** 10.1038/s41377-020-0249-4

**Published:** 2020-01-28

**Authors:** Dashan Dong, Xiaoshuai Huang, Liuju Li, Heng Mao, Yanquan Mo, Guangyi Zhang, Zhe Zhang, Jiayu Shen, Wei Liu, Zeming Wu, Guanghui Liu, Yanmei Liu, Hong Yang, Qihuang Gong, Kebin Shi, Liangyi Chen

**Affiliations:** 1 State Key Laboratory for Mesoscopic Physics and Frontiers Science Center for Nano-optoelectronics, School of Physics, Peking University, Beijing, 100871 China; 2 Collaborative Innovation Center of Extreme Optics, Shanxi University, Taiyuan, Shanxi 030006 China; 3 State Key Laboratory of Membrane Biology, Beijing Key Laboratory of Cardiometabolic Molecular Medicine, Institute of Molecular Medicine, Peking University, Beijing, 100871 China; 4 School of Mathematical Sciences, Peking University, Beijing, 100871 China; 5 School of Software and Microelectronics, Peking University, Beijing, 100871 China; 6 State Key Laboratory of Stem Cell and Reproductive Biology, Institute of Zoology, Chinese Academy of Sciences, Beijing, 100101 China; 7 University of Chinese Academy of Sciences, Beijing, 100049 China; 8 National Laboratory of Biomacromolecules, Chinese Academy of Sciences Center for Excellence in Biomacromolecules, Institute of Biophysics, Beijing, 100101 China; 9 Institute for Brain Research and Rehabilitation (IBRR), Guangdong Key Laboratory of Mental Health and Cognitive Science, South China Normal University, Guangzhou, China; 10 Collaborative Innovation Center of Quantum Matter, Peking University, Beijing, 100871 China; 11 PKU-IDG/McGovern Institute for Brain Research, Beijing, 100871 China

**Keywords:** Biophotonics, Interference microscopy

## Abstract

The emergence of super-resolution (SR) fluorescence microscopy has rejuvenated the search for new cellular sub-structures. However, SR fluorescence microscopy achieves high contrast at the expense of a holistic view of the interacting partners and surrounding environment. Thus, we developed SR fluorescence-assisted diffraction computational tomography (SR-FACT), which combines label-free three-dimensional optical diffraction tomography (ODT) with two-dimensional fluorescence Hessian structured illumination microscopy. The ODT module is capable of resolving the mitochondria, lipid droplets, the nuclear membrane, chromosomes, the tubular endoplasmic reticulum, and lysosomes. Using dual-mode correlated live-cell imaging for a prolonged period of time, we observed novel subcellular structures named dark-vacuole bodies, the majority of which originate from densely populated perinuclear regions, and intensively interact with organelles such as the mitochondria and the nuclear membrane before ultimately collapsing into the plasma membrane. This work demonstrates the unique capabilities of SR-FACT, which suggests its wide applicability in cell biology in general.

## Microscopy: New tool furthers our understanding of living cells

Scientists from China have developed a new microscopy tool that could help to further expand our understanding of cell biological processes in live cells. Although the emergence of superresolution (SR) fluorescence microscopy is revolutionising the life sciences, the technique can only be used to observe a handful of biomolecules simultaneously and cannot provide a holistic map of the cellular landscape. Furthermore, the imaging of live cells in three dimensions (3D) over time also remains very challenging. By combining two different techniques, a team of Chinese researchers led by Liangyi Chen and Kebin Shi from Peking University has created a novel dual-mode high-speed SR microscopy technique. The tool is capable of revealing previously unseen sub-cellular structures and dynamic processes, allowing both the cellular landscape and molecular identity in live cells to be observed in 3D over prolonged periods.

## Introduction

In terms of the search for new structures and dynamics, the emergence of super-resolution (SR) fluorescence microscopy techniques in the 21st century is expected to reshape all aspects of modern life science^1^. However, limited by the broad emission spectrum of fluorophores and excessive phototoxicity, SR fluorescence microscopy can only be used to highlight a handful of biomolecules simultaneously and is incapable of providing a holistic map of the cellular environment and landscape. While electron microscopy can be combined to reveal the cellular landscape information in addition to molecular details provided by fluorescence microscopy^2^, such endpoint experiments only provide snapshots of dead cells and cannot follow the dynamic processes in live cells. Another ultimate challenge is how to conduct live-cell SR imaging in three dimensions (3D) for a prolonged period of time. For example, in addition to the slow speed of physically adjusting the axial position, 3D-structured illumination microscopy (3D-SIM) needs to acquire a number of raw images that is at least an order of magnitude greater than that of 2D-SIM to extract the SR information in the *Z*-axis, and is thus unlikely to be used for long-term imaging.

On the other hand, because different cellular organelles exhibit different refractive index (RI) values^3^, they may cause the scattering of incident light in live cells, which can be measured by phase-contrast microscopy techniques such as differential interference contrast microscopy^4^. Recently, by using wide-field digital holograms and tomographic illumination, various types of optical diffraction tomography (ODT) have been developed to extend quantitative phase imaging to three dimensions^5–8^. However, all previous ODT microscopes have mainly focused on improving the theoretical spatial resolution at the expense of reduced temporal resolution. As a result, the image contrast and resolution of fast-moving structures such as lysosomes will be compromised in live-cell experiments (detailed in Supplementary Notes 1.1, Supplementary Fig. S1). Therefore, despite the claimed ~100-nm spatial resolution, which should be sufficient to resolve most organelles in cells, lipid droplets (LDs), chromosomes, and mitochondria are the organelles seen by the current ODT microscopes^5,6,9,10^. In addition, a lack of molecular specificity in label-free ODT microscopy also hinders the interpretation of imaging results. Although two-dimensional phase-contrast microscopy can be combined with fluorescent confocal microscopy for live-cell studies, the low resolution of label-free images prevents the accurate prediction of the organelles within cells^11,12^. Therefore, all previous dual-mode imaging microscopy techniques failed when used for time-lapsed correlated SR imaging in live cells^11–13^, which is the focus of this study.

Thus, we report a dual-mode high-speed SR microscopy technique termed SR fluorescence-assisted diffraction computational tomography (SR-FACT) that visualizes both the cellular landscape and the molecular identity of live cells. A new algorithm termed the vector iterative search algorithm (VISA) was developed to minimize 3D imaging reconstruction errors under high-speed kHz-rate tomographic scanning scheme. As a result, SR-FACT can simultaneously utilize a maximal imaging speed to capture dynamics in live cells and to maintain sufficient photon flux for maximal sensitivity. In the reported SR-FACT system, the ODT module achieved an ~200-nm lateral resolution at a volumetric imaging speed of 0.8 Hz (40 × 40 × 20 μm^3^). Hessian 2D-SIM, which allows SR imaging at a fraction of the photon dose used by conventional SIM^14^, was used to guide the interpretation of structures observed by the ODT module. By performing dual-mode correlated imaging in COS-7 cells, we resolved six known organelles without labeling: the tubular endoplasmic reticulum (ER), mitochondria, late endosomes/lysosomes (LEs/LYs), LDs, the nuclear membrane, and chromosomes. All these data highlight the unique advantage of SR-FACT in studying the organelle interactome. Moreover, we also observed vacuolated structures with neutral pH that contained mostly liquid in the lumen. Hour-long time-lapsed live-cell SR imaging in combination with quantitative analysis reveals the unconventional trafficking routes and indispensable roles of vacuoles in organizing the organelle interactome, all of which suggest that they represent previously unappreciated organelles.

## Results

### Experimental implementation and image reconstruction of SR-FACT

**Fig. 1:**
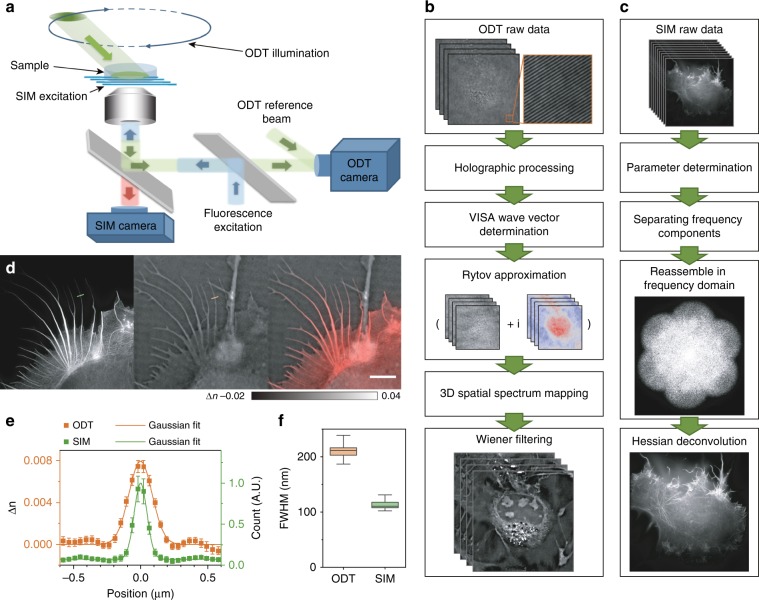
Hardware implementation, algorithm pipeline, and resolution benchmarking of SR-FACT. **a** Schematic diagram of the SR-FACT setup. **b** Flow chart of the ODT reconstruction algorithm. **c** Flow chart of the SIM reconstruction algorithm. **d** Representative example of a COS-7 cell labeled with LifeAct-EGFP imaged with SR-FACT. Scale bar, 5 μm. **e** Average intensity profiles of RIs (orange) and fluorescence (green) along lines orthogonal to the actin filaments (with the same filament at 50 time points); a representative example is shown in (**d**). **f** Average full width at half maximum (FWHM) values of the profiles orthogonal to the actin filament labeled by the lines in (**d**) measured at 50 different time points. Center lines, medians; limits, 75 and 25%; whiskers, maximum and minimum.

To increase the temporal resolution while measuring the scattered optical field of light passing through biological samples due to the inhomogeneous distribution of the RI, we built an off-axis holographic ODT system based on a commercial microscope (IX73, Olympus) equipped with a galvo-mirror scanning mechanism. A schematic of the hardware setup is shown in Fig. 1a, while the full setup is shown in Supplementary Fig. S2 and explained in detail in Supplementary Note 2. In brief, we used a 561-nm single longitudinal-mode laser (MSL-FN-561-50 mW, Changchun New Industries Optoelectronics Technology) as the illumination source for ODT microscopy. The 561-nm light was divided into two beams by a polarization-dependent beam splitter (CCM1-PBS251, Thorlabs), of which one beam was used to illuminate the sample on the microscope stage, while the other served as a reference. The illuminating beam was controlled by galvo mirrors (2 × GVS211/M, Thorlabs) focused on the back focal plane of the water immersion objective (60×/1.0 W, LUMPlanFLN, Olympus), passed through the sample and the detection objective (100×/1.45 Oil, ApoN, Olympus) at a tilted angle, and finally combined with the reference beam to generate a hologram on the sCMOS camera (ORCA-Flash 4.0 V3, Hamamatsu), which provided sufficient total photon flux within 50 μs of exposure time. Because each image only captured information from a portion of the spatial frequency domain of the sample, we used a delicate time sequence to adjust the position of the focal point on the back focal plane of the illumination objective lens to record 240 raw holograms at different illumination angles (Supplementary Fig. S3) and reconstructed the spatial frequency for the whole volume thereafter.

The pipeline for the ODT image reconstruction is shown in Fig. 1b and is explained in detail in Supplementary Fig. S4. By conducting Fourier transformation of the raw images, spectrum filtering in the Fourier domain, and inverse Fourier transformation, we first extracted the amplitudes and phases from the experimental images at different time points. Due to the high scanning speed (~200 Hz) and the long recording time, we found that the mechanical wobbling and instability of the galvo mirrors led to deviation of the illumination wave vectors from the designated angles (Supplementary Fig. S5), which significantly compromised both the contrast and the resolution of the reconstructed images. To address this problem, we designed the VISA algorithm, which precisely determines the actual illumination wave vectors and minimizes the residue slope of the unwrapped phase distribution extracted from the holographic recordings (Fig. 1b; Supplementary Figs. S6, S7). In the third step, we used the first-order Rytov approximation (Eq. (7) in Supplementary Note 3) to estimate the scattering of light passing through live cells according to the different RIs exhibited by organelles. The amplitude and unwrapped phases derived from the 2D holograms were then recast in the 3D frequency domain. Because each illumination angle only provided a portion of the full scattering field of the cell, we stitched together the Rytov field distributions obtained at different illumination angles in the spatial frequency domain to perform the total field mapping. Finally, based on the measured coherent transfer function of the microscope (Supplementary Fig. S8), we used Wiener filtering (Eq. (10) in Supplementary Note 3) to reconstruct the 3D distribution of the RIs within the cell.

In the SIM configuration, we used a 488-nm single longitudinal-mode laser (Sapphire 488LP-200, Coherent) as the illumination source and an acoustic optical tunable filter (AOTF, AA Opto-Electronic, France) to adjust the illumination power. To generate and switch the various excitation patterns, we used a ferroelectric liquid crystal on a silicon spatial light modulator (SLM) (SXGA-3DM, Fourth Dimension Display) with a high frame rate. To maximize the modulation contrast of the excitation, we used a liquid crystal variable retarder (LVR-200-VIS-1L-TSC, Meadowlark) combined with a quarter-wave plate to rotate the polarization of the input beams to S-polarization. For the emission path, we designed a synchronization paradigm that efficiently coordinated the pattern generation of the SLM and the camera readout interval and used an sCMOS camera with 82% peak quantum efficiency (ORCA-Flash 4.0 V2, Hamamatsu) to detect the fluorescent emission (Fig. 1a; Supplementary Fig. S2). The steps used for the Hessian SIM reconstruction are outlined in Fig. 1c, while the detailed procedure can be found in our previous paper^14,15^.

To ensure the ultrafast sampling of raw images and accurate switching between the ODT and SIM, we designed an intricate time sequence to synchronize the ODT and the SIM data acquisition (Supplementary Fig. S3). The whole cycle of ODT and SIM acquisition took 1.49 s, which is fast enough to allow the same structure in a live cell to be examined alternatively by the two modalities. For example, by examining the same actin filament in filopodia in a COS-7 cell, we evaluated the resolutions of both modalities for the same filament structures. Using a Gaussian function to fit the intensity profiles of LifeAct-EGFP along cross-sections of the actin filament, we found that the resolution of Hessian SIM is ~100 nm (Fig. 1d–f), which is consistent with previous results^14^. On the other hand, the scattered field in the ODT essentially caused a horizontal frequency shift in the detection frequency domain, which increased the lateral resolution. The full width at half maximum (FWHM) of the actin filament measured by our ODT microscope was ~200 nm (Fig. 1d–f), which exceeded the conventional lateral resolution obtained by fluorescence microscopy at this wavelength and was consistent with the theoretical prediction given in Supplementary Note 7 (Supplementary Fig. S9).

### Visualization of structures and dynamics associated with nuclear membrane formation and disintegration during mitosis

**Fig. 2:**
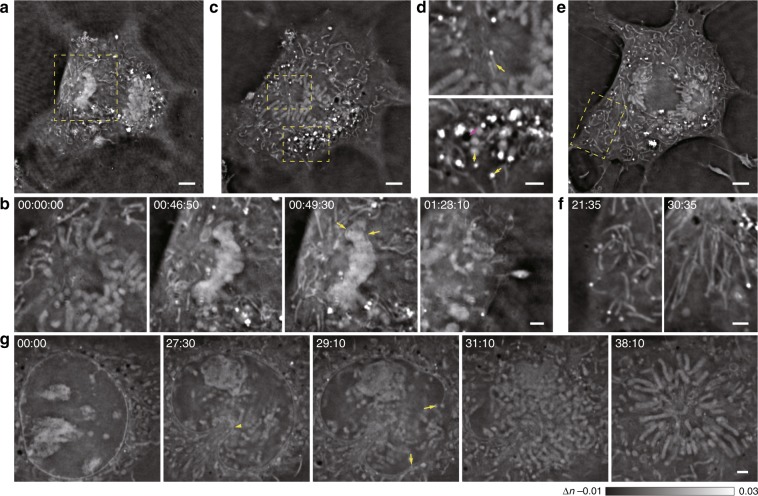
Label-free visualization of the complete division process of a COS-7 cell in three dimensions, in which various subcellular structures are observed. Cell division followed by late nuclear membrane emergence in one representative example of three similar replicates is shown in **a**–**f**, and the disintegration of the nuclear membrane in another representative cell is shown in **g**. **a** One Z plane of a COS-7 cell is shown at a time point of 00:49:30. **b** The region enclosed by the dashed yellow box in (**a**) is enlarged and shown at four different time points, in which the material condensation (middle left), emergence of the membrane structure (middle right, arrows), and final chromatin foci formation (right) are shown. **c**, **d** Another Z plane of the COS-7 cell (0.86 μm below the plane in **a**) is shown at the time point of 00:00:00. **c** The region enclosed by the dashed yellow box was enlarged and is shown in (**d**), in which smaller filament structures (upper arrow), bright puncta (lower yellow arrow), large dim vesicles (lower yellow arrowhead), and black vacuole-like vesicles (lower magenta arrow) are shown. **e**, **f** The third Z plane of the COS-7 cell (1.72 μm below the plane in **a**) is shown at the time point of 00:43:10. **e** The region enclosed by the dashed red box is enlarged and shown at two time points (**f**), before (left) and after (right) cytokinesis. Freely spanning and twisted worm-like tubular structures are shown in the left panel, and are aligned along the nucleus–cell exterior spindles in the right panel. **g** A zoomed-in image of another cell shows one plane of the nuclear region before cell division. Five different time points are shown, with clear visualization of the cell nucleus and associated nucleolar structures (0’00”), the deformation of one region in the nuclear membrane (the arrowhead) by many incoming cellular structures (27’30”), the initial rupture of the nuclear membrane in other regions (arrows, 29’10”), emergence of chromosomes (31’10”), and alignment of chromosomes into petal-like arrays (38’10”). Scale bars: (**a**, **c**, **e**) 5 µm and (**b**, **d**, **f**, **g**) 2 µm.

Our ODT microscope possessed high volumetric imaging capacity, high spatiotemporal resolution, and low phototoxicity, which enabled the monitoring of structures and dynamics in cellular processes that are prone to phototoxicity, such as mitosis, over a prolonged period of time. As a representative example, shown in Supplementary Video 1, we observed doublet structures that resembled chromosomes in the nuclear region of a dividing cell. During mitosis, the chromosomes were first pulled apart and then formed two large, closely connected and highly dense patches (Fig. 2a)^16^. Next, the formation of membrane-like structures could be observed as early as when the condensed structure began to disintegrate into different clusters, which were optically dense compared with the surrounding environment (Fig. 2b). In the cytosol, we also observed various structures with different shapes, densities, and dynamics. For example, intricate filament structures at the centrosome position could be observed, while bright vesicular structures, large dim vesicles, and black vacuole-like vesicles were clustered in other regions (Fig. 2c, d). We observed worm-like tubular structures that freely spanned and twisted most of the time, becoming aligned along the nucleus–cell exterior spindles during cytokinesis (Fig. 2e, f). In another example (Fig. 2g; Supplementary Video 2), the cell nucleus and associated nucleoli structures rotated, which was followed by the attachment and deformation of one region in the nucleus membrane caused by many incoming cellular organelles^17^. Then, disintegration of the nuclear membrane at the region opposite to the initial invaginated site was observed, followed by the emergence of chromosomal structures and, finally, the alignment of these structures into petal-like arrays. Despite the distinct spatiotemporal dynamics of subcellular structures revealed by the ODT module, their identities remained to be explored by colocalization with existing fluorescent organelle markers by using simultaneous Hessian SIM imaging.

### Confirmed visualization of six classical organelles from ODT images

**Fig. 3:**
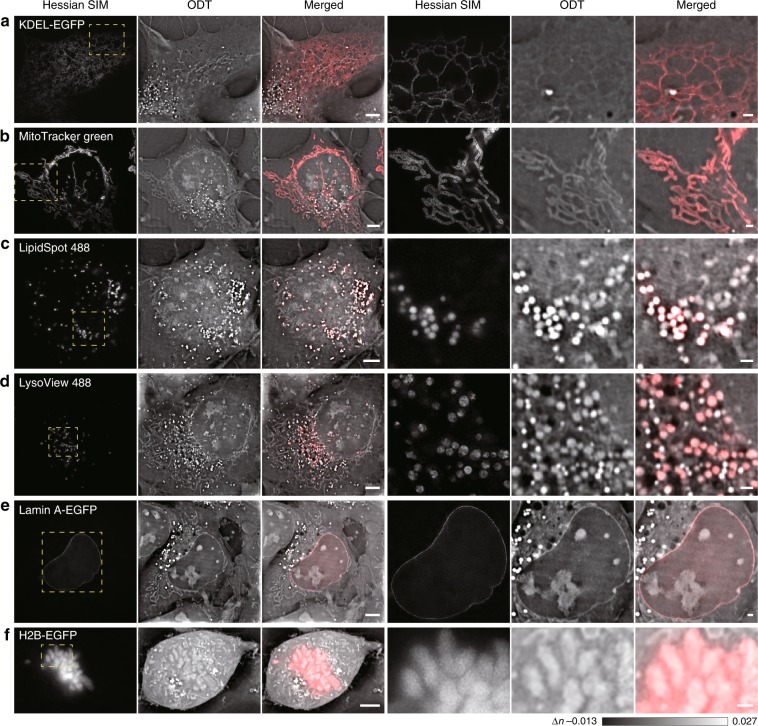
Confirmed visualization of six conventional cellular organelles from ODT images. The regions enclosed by the dashed yellow boxes on the left are enlarged on the right. **a** Observation of tubular ER structures in a live COS-7 cell from ODT images as confirmed by colocalization with KDEL-EGFP-labeled structures in the Hessian SIM channel. **b** Observation of mitochondria in a live COS-7 cell from ODT images, as confirmed by colocalization with MitoTracker Green-labeled structures in the Hessian SIM channel. **c** Observation of lipid droplets in a live COS-7 cell from ODT images, as confirmed by colocalization with LipidSpot 488-labeled structures in the Hessian SIM channel. **d** Observation of lysosomes in a live COS-7 cell from ODT images, as confirmed by colocalization with LysoView 488-labeled structures in the Hessian SIM channel. **e** Observation of the nuclear membrane in a live COS-7 cell from ODT images, as confirmed by colocalization with the Lamin A-EGFP-labeled nuclear membrane in the Hessian SIM channel. **f** Observation of chromosomes in a live COS-7 cell from ODT images, as confirmed by colocalization with H2B-EGFP-labeled structures in the Hessian SIM channel. Scale bars, 5 µm (left) and 1 µm (right).

The ER is the most prominent organelle and the hub of the organelle interactome^18,19^, and this organelle has never been resolved by live-cell imaging without labeling^6,20,21^. Due to the improved spatiotemporal sensitivity, we observed vigorous movement of dim tubular structures resembling the ER in a resting COS-7 cell, which was confirmed by the perfect colocalization of these structures with KDEL-EGFP-labeled structures in the SIM images (Fig. 3a; Supplementary Video 3). On the other hand, the worm-like, bent and twisted segments were confirmed to be mitochondria, as they perfectly overlapped with structures labeled by MitoTracker Green, a mitochondria-specific marker (Fig. 3b; Supplementary Video 4). Although the structural dynamics of the inner cristae within different mitochondria could be resolved by 2D Hessian SIM, this method provided information extracted from only one axial plane. In contrast, the label-free ODT module provided 3D maps of the total mitochondria within a cell, which covered an area that was ~3 times the maximal area of mitochondria that can be detected within an axial volume of ~0.86 μm (approximating the axial volume of 2D-SIM, Supplementary Fig. S10). In addition, compared with the susceptibility of the mitochondria to the excessive phototoxicity generated from labeled fluorescent indicators during imaging^22^, ODT imaging confers no photobleaching or phototoxicity, as demonstrated by more than 1 h of 3D imaging of cells without perturbing the division process (Fig. 2).

The brightest cellular structures in the ODT images possibly resembled LDs with high RIs, as previously suggested^6^. By showing their colocalization with structures labeled with LipidSpot 488, a dye that specifically accumulates in LDs, we confirmed that these highly scattering vesicles were indeed LDs (Fig. 3c; Supplementary Video 5). Compared with LDs, the larger and less bright vesicular structures were confirmed to be LEs/LYs, as these structures were labeled by LysoView 488, an acidic LE/LY indicator (Fig. 3d; Supplementary Video 6).

Finally, we explored the identities of structures in the cell nucleus. The continuous membrane structure enclosing the nucleus in ODT images was the nuclear membrane, since it colocalized well with lamin A-EGFP, which targets the nuclear lamina (Fig. 3e; Supplementary Video 7). The multiple bright, irregular structures in the nucleus were chromosomes, as indicated by their colocalization with H2B-EGFP-labeled fluorescent structures (Fig. 3f; Supplementary Video 8). This experiment also revealed the nonnegligible phototoxicity induced by fluorescence imaging, as cells imaged with Hessian SIM failed to form spindle-shaped chromosomes and were arrested at late prophase (Supplementary Fig. S11, Supplementary Video 9), which was in sharp contrast to the completion of cell division observed in cells imaged only with the ODT modality (Fig. 2). We also fluorescently labeled other organelles, such as the Golgi and peroxisomes, but failed to identify corresponding structures in the ODT images (Supplementary Fig. S12). Thus, these organelles may have RIs that are not markedly different from that of the cytosol and, thus, are invisible in ODT images.

In addition to these conventional organelles, we also observed dark vacuoles that exhibited an RI that was even lower than that of the cytosol in COS-7 cells (Fig. 2d). Because ODT measures the spatiotemporal distribution of mass density within live cells^9,23,24^, these vacuolated structures contained much less material than the cytosol and resembled vacuoles in plants and yeasts^25,26^. However, in contrast to the 5–10-μm central acidic vacuoles in plants and yeasts, the vacuole-like vesicles were small (1.56 ± 0.01 μm, *n* = 5162), numerous (43 ± 2, one plane of COS-7 cells, 119 cells), and not labeled by fluorescent dyes targeted to acidic compartments (Fig. 3d). In fact, vacuoles in yeast observed under the same ODT microscope appeared larger and less dark than the vacuolated vesicles in mammalian cells (Supplementary Fig. S13). Therefore, we named these structures dark-vacuole bodies (DBs). Overall, we established that, without labeling and with minimal phototoxicity, SR-FACT can reveal six conventional organelles (ER, mitochondria, LDs, LEs/LYs, the nuclear membrane, and chromosomes) and one possible previously unappreciated organelle in 3D within live cells.

### SR-FACT reveals the one-to-one contacts of the mitochondria with other organelles

Organelles are cellular compartments that preserve the local imprinting of molecules and signals and exchange information and materials with other organelles upon the transient formation of organelle contacts, which are crucial to many cellular functions and behaviors^18,27^. From an evolutionary perspective, the ER and mitochondria are both ancient eukaryotic endo-membrane systems^28^. However, in contrast to numerous studies that have focused on the role of the ER in coordinating the organelle interactome^19^, relatively few studies have been devoted to the systematic evaluation of the interactions of mitochondria with different organelles^28^. One bottleneck could be the phototoxicity associated with the fluorescence imaging of the mitochondria^22^. With minimal phototoxicity, we could continuously monitor mitochondria in live cells for a long period of time, and we found that mitochondria actively change shapes, positions, fates, and possibly functions according to their interactions with other organelles.

**Fig. 4:**
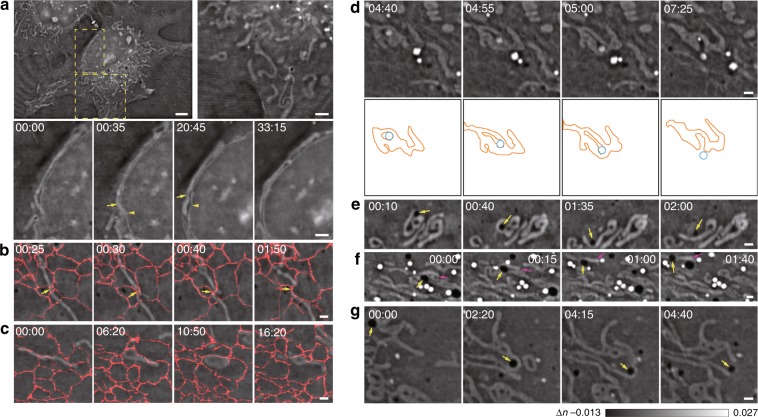
Different dynamics of mitochondria interacting with other organelles. **a** Representative examples of different shapes of cytosolic mitochondria and mitochondria associated with the nuclear membrane. In contrast to cytosolic mitochondria that have various shapes, lengths, and orientations (enlarged in the right panel), long mitochondria associated with the nuclear membrane for half an hour (enlarged and shown as a montage in the lower panel) and exhibited dynamic morphological changes with reciprocal movement of the nuclear membrane (arrows and arrowheads). **b**, **c** Two representative distinct examples of ER–mitochondria contacts. One contact (indicated by the arrows) led to scission of the mitochondrion (**b**), while the other contact led to the lateral expansion of the mitochondrion (**c**). We overlaid the fluorescent signals of KDEL-EGFP on these images to better present the data. **d** A representative example of a lysosome that was initially surrounded by the same mitochondrion, which caused fission and then moved out of the mitochondrion. Montages of actual images (top) and corresponding schematic diagrams (bottom) are shown. **e**–**g** Representative examples of three modes of mitochondrion–DB contacts. The collision of DBs (arrows) with the mitochondrion changed the mitochondrial morphology (**e**), caused the mitochondrion (magenta arrows) to move along with the DB (highlighted by yellow arrows in **f**), or caused fission (arrows) of the interacting mitochondrion (**g**). Scale bars: (**a**) 5 μm and 2 μm (zoom in); (**b**–**g**) 2 μm.

For example, compared with irregular mitochondria that were randomly distributed in the cytosol, mitochondria that were closely and stably associated with the nuclear membrane were long and directly apposed to the nuclear membrane (Fig. 4a; Supplementary Video 10). In fact, dynamic changes in mitochondrial morphology were accompanied by reciprocal changes in the nuclear membrane (Fig. 4a). Therefore, these nucleus-interacting mitochondria may perform important functions adjacent to the nucleus, such as prowering mRNA export from the nucleus to the cytoplasm^29,30^. On the other hand, although the ER shares a common boundary with the nuclear membrane, ER–mitochondria interactions are apparently different. Consistent with the results of most studies^28,31,32^, we detected the scission of mitochondria guided by their interactions with the ER (Fig. 4b; Supplementary Video 11). Unexpectedly, we also observed ER–mitochondria interactions that were not reported previously; the pulling by the ER of both sides of the mitochondrion causes the latter to expand into a sheet (Fig. 4c; Supplementary Video 12), and this may involve proteins, lipids, and other tethers that are distinct from those observed in previous reports. In addition to the mitochondria, another organelle that is important for cellular energy handling are LDs, which have been reported to interact with the mitochondria in a variety of cell types^33,34^. We observed mostly short-term interactions between the mitochondria and LDs in resting cells. For example, after an LD collided with a mitochondrion, it was rapidly pushed back, and the morphologies of the LD and the mitochondrion were not significantly altered (Fig. 4d; Supplementary Video 13). In comparison, when an LE/LY that was initially surrounded by the same mitochondrion started to move, the surrounding mitochondrial regions that were in contact with the LE/LY withdrew, while the shape of the regions that did not form such contacts remained unchanged (Fig. 4d). The same process continued until the LE/LY was completely outside the mitochondrion, which indicated the possible role of LEs/LYs in mediating mitochondrial fission, which is consistent with the results of a previous work^35^. Interestingly, DBs also closely interacted with mitochondria in a different manner. For example, one collided with a mitochondrion and changed the shape of the latter (Fig. 4e; Supplementary Video 14), while another carried the mitochondrion with itself (Fig. 4f; Supplementary Video 15); in some cases, contact of a DB with a mitochondrion also caused mitochondrial fission (Fig. 4g; Supplementary Video 16). Therefore, instead of forming a continuous network to interact with other organelles such as the ER, mitochondria adopt a “one-to-one” type of contact, by which an individual mitochondrion is customized to interact with different organelles under various conditions.

### Hour-long SR-FACT imaging reveals the trafficking of DBs and enables their interactions with other organelles to be quantified

**Fig. 5:**
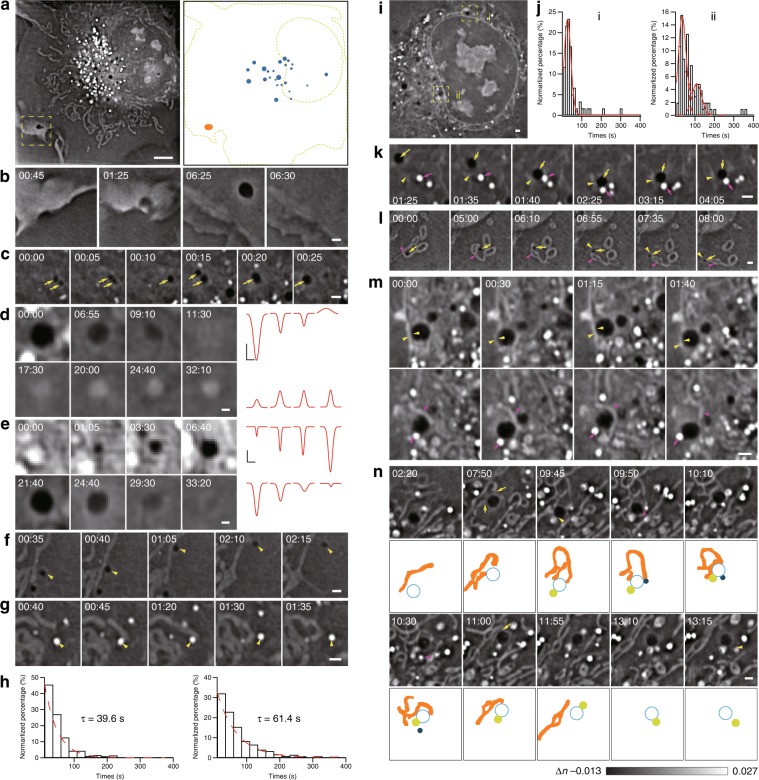
Visualization of the trafficking routes of DBs and their role in organizing the organelle interactome. **a** One Z plane of the COS-7 cell is shown at the time point of 00:49:30 (left), while the corresponding schematic diagram shows the distributions of DBs close to the nuclear membrane (blue) and the plasma membrane (orange, right). **b** A representative example of a large DB formed in the cell periphery due to pinocytosis. **c** A representative example of two sequential DB–DB fusion events. Arrows indicate DBs. **d** A representative example of the transformation of the DB into an LE/LY in a COS-7 cell is shown on the left, while the corresponding intensity profiles at different time points approximated by the Gaussian function are shown on the right. **e** A representative example of the biogenesis of a DB in the region close to the nuclear membrane (top), followed by fusion of the DB to the plasma membrane ~27 min later (bottom). Montages of the DB at different time points are shown on the left, while the corresponding intensity profiles at different time points approximated by the Gaussian function are shown on the right. **f**, **g** A representative example of a DB–mitochondrion (**f**, the arrowhead indicates the DB) or LD–mitochondrion (**g**, the arrowhead indicates the LD) contact. **h** Distribution of the durations of LD–mitochondrion contacts (left) and durations of DB–mitochondrion contacts (right). **i** One cell with DB–nuclear membrane contacts in sparsely (i) and densely (ii) populated perinuclear regions. **j** Histograms of contact times between DBs and the nuclear membrane in sparsely (left) and densely (right) populated regions. **k** A representative example of a multiorganelle complex formed by collision of a DB (yellow arrows) with a contact formed by the tubular ER (yellow arrowheads) and an LD (magenta arrows). **l** Another representative example of a multiorganelle complex formed by a DB (yellow arrows), a mitochondrion (magenta arrowheads), and the tubular ER (yellow arrowheads). **m** A representative example of a DB bridging different organelles. The same DB (yellow arrows) interacted with the nuclear membrane (yellow arrowheads) in one Z plane (upper panel) and simultaneously with both an LD (magenta arrows) and a mitochondrion (magenta arrowheads) in the Z plane 0.68 μm away (lower panel). **n** Montage of a representative example of a DB that sequentially interacted with a mitochondrion (yellow arrows), an LY (yellow arrowhead), and an LD (magenta arrowhead) to form the DB–mitochondrion–LD–LY quaternary complex, followed by the disassociation of the LD, the mitochondrion and the LY one-by-one. Schematic diagrams are shown below the ODT images to better demonstrate the process. Scale bars: (**a**) 5 μm and (**b**–**g**, **i**, **k**–**n**) 1 μm.

To explore the identities of DBs, we first visualized their biogenesis and disappearance by long-term live-cell imaging. While large vacuoles could originate from micropinocytosis close to the plasma membrane, most of the normal-sized vacuoles emerged at regions close to the nuclear membrane (Fig. 5a, b; Supplementary Video 17). Within their lifetimes in live cells, these vesicles also fused with each other to grow in size (Fig. 5c; Supplementary Video 18). Ultimately, while a minority of DBs slowly transformed into LEs/LYs (3 out of 26, from 2 cells) (Fig. 5d; Supplementary Video 19), the majority of the DBs (23 out of 26, from 2 cells) collapsed into the plasma membrane (Fig. 5e; Supplementary Video 20).

Next, we quantitatively measured the duration of DB–mitochondrion contact, which followed an exponential distribution with an ensemble time constant of ~61 s. As a control, the mean duration of the LD–mitochondrion contact was determined and found to be significantly shorter (~37 s), which was consistent with previously reported results^34^ (Fig. 5f–h; Supplementary Videos 21–22). These data suggest the occurrence of strong interactions between DBs and mitochondria. Intriguingly, we also observed that DBs frequently interacted with the nuclear membrane (Fig. 5i). We calculated the time intervals of DE–nuclear membrane contacts at perinuclear regions with sparsely (i) or densely (ii) distributed structures (Fig. 5j). Intriguingly, in both regions, we found that duration times could best be fitted by Gaussian distributions, which suggested that the DB–nuclear membrane contacts were governed by multiple interacting processes. In addition, because an additional peak at ~105 s was present in the histogram of the contact times between DBs and the nuclear membrane in region ii, the interactions in region ii were shown to be much stronger than those in region i.

Finally, we found that DBs frequently played a central role in the formation of multiorganelle complexes. For example, both a DB and an LD were attached to different sides of one ER tubule to form a multiorganelle complex for more than 2 mins before detachment (Fig. 5k; Supplementary Video 23); a DB wrapped within ER tubules could also attach to a mitochondrion and linger alongside the mitochondrion for at least one minute (Fig. 5i; Supplementary Video 24). The DB itself could also connect to multiple organelles simultaneously. For example, one DB was firmly attached to the nuclear membrane in one focal plane, and time-dependent and reciprocal changes in the morphology of both organelles were observed; in another focal plane 0.68 μm away, the same DB simultaneously interacted with an LD and a mitochondrion on two different sides (Fig. 5m; Supplementary Videos 25–26). In another example, one DB sequentially established contacts with a mitochondrion (7’50”), an LY (9’45”), and an LD (9’50”) to form one multiorganelle complex, which lasted for 40 s before the dissociation of the LD (10’30”), the mitochondrion (11’00”), and the LY (13’15”) (Fig. 5n; Supplementary Video 27). Overall, these data suggest that DBs may serve as a central hub in coordinating the organelle interactome and organizing multiorganelle complexes.

DBs were identified in a variety of cells, including human fibroblasts, umbilical vein endothelial cells, rat insulinoma INS-1 cells, and mouse dorsal root ganglion neurons (Supplementary Fig. S14). Interestingly, in different types of ageing human mesenchymal stem cells (hMSCs)^36,37^, DBs were associated with a severe ageing phenotype (Supplementary Fig. S15, Supplementary Videos 28–31). In addition, starvation triggered a reduction in the average number of DBs in COS-7 cells (Supplementary Fig. S16). Taken together, these data suggest the general role of DBs in normal cell function.

### Dual-mode correlative imaging revealed the overall membrane component profile of DBs

**Fig. 6:**
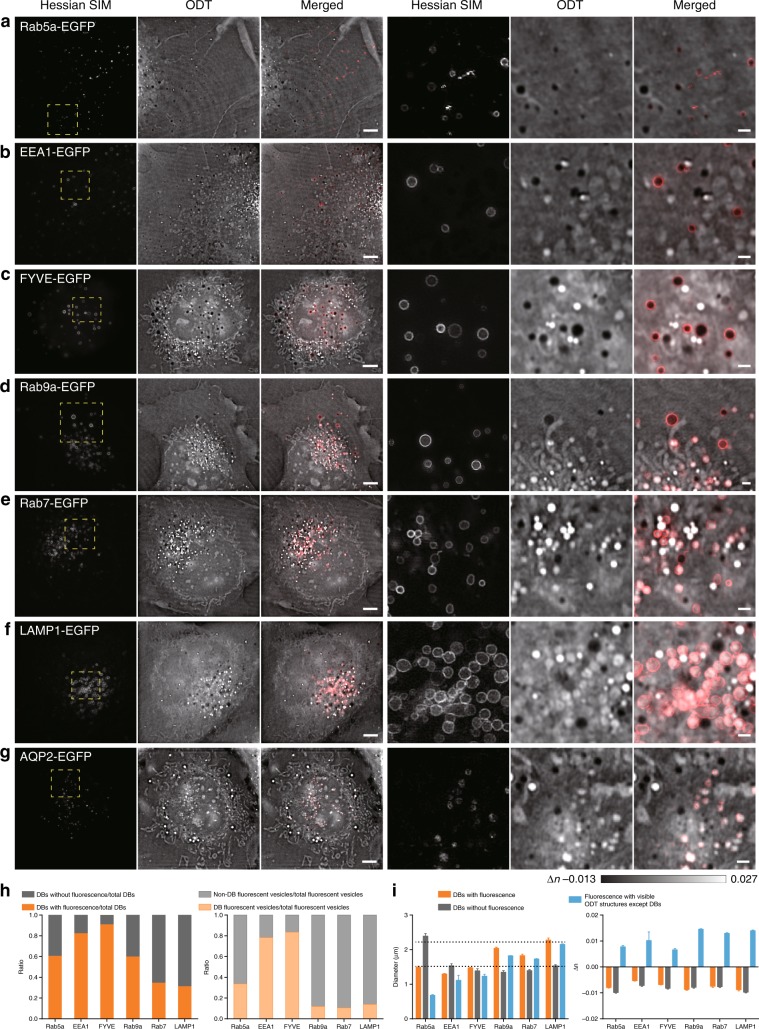
DBs exhibited an overall distinct profile from that of conventional endosomal compartments, despite some shared protein markers. The regions enclosed by the dashed yellow boxes on the left are enlarged on the right. **a**–**f** Representative examples of the colocalization of DBs with Rab5a-EGFP- (**a**), EEA1-EGFP- (**b**), FYVE-EGFP- (**c**), Rab9a-EGFP- (**d**), Rab7-EGFP- (**e**), LAMP1-EGFP- (**f**), and AQP2-EGFP- (**g**) labeled vesicles. **h** Left: proportions of DBs associated with fluorescent Rab5a/EEA1/FYVE/Rab9a/Rab7/LAMP1 within the total pool of DBs are shown in orange, while those without fluorescence labeling are shown in gray. Right: proportions of DBs associated with Rab5a/EEA1/FYVE/Rab9a/Rab7/LAMP1 within the total pools of the respective fluorescent vesicles are shown in orange, while those of non-DB fluorescent vesicles within the total pools of the respective fluorescent vesicles are shown in gray. **i** Left: diameters of DBs with (orange) and without (gray) fluorescent labeling and fluorescent vesicles with visible ODT structures except DBs (blue). Right: differences in RIs between cellular structures and their surrounding environments, which include DBs with (orange) and without (gray) fluorescent labeling and fluorescent vesicles with visible ODT structures, except DBs (blue). Scale bars, (**a**–**g**) 5 μm (left) and 1 μm (right).

By examining cells with different exogenously expressed fluorescent markers (Rab5a/EEA1/FYVE/Rab9a/Rab7/LAMP1), we systematically analyzed proteins and lipids residing on the DB membrane (Fig. 6a–g). A total of 61 ± 3% of DBs (average diameter ~1.5 μm, as measured by the outer fluorescent rings) were associated with the EE marker Rab5a-EGFP, while a large number of Rab5a-EGFP-labeled vesicles exhibited RI values higher than those of DBs (66 ± 4%, Fig. 6h). Further downstream of the endocytic trafficking pathway, the DBs colocalized with LE/LY markers increased in size (average diameter 1.8–2.3 μm, Fig. 6i). While Rab9a-EGFP labeled 60 ± 6% of all vacuolated vesicles, these vesicles constituted only 12 ± 1% of all Rab9a-EGFP-labeled vesicles. Approximately 31–35% of vacuolated vesicles were labeled with Rab7-EGFP or LAMP1-EGFP, and these colocalized vesicles represented a minor proportion of Rab7-EGFP/LAMP1-EGFP vesicles (~11–14%). Because Rab7 and LAMP1 are more exclusively associated with LE/LY than Rab9a^38,39^, we propose that 31–35% of DBs may share the characteristics of LEs/LYs. Likewise, the 61 ± 3% of DBs that overlapped with Rab5a-EGFP-labeled structures may correspond to the population that is similar to EEs. The overlap of 82–91% of vacuolated vesicles with EEA1-EGFP- and FYVE-EGFP-labeled structures indicated the enrichment of phosphatidylinositol 3-phosphate lipids on DBs.

We also examined the colocalization between DBs and autophagosomes labeled by LC3-EGFP. Although ring-shaped LC3-EGFP occasionally colocalized with the outer membranes of either LEs/LYs or large DBs (Supplementary Fig. S17c, d), the majority of the LC3-EGFP formed puncta that either did not overlap with clear ODT structures (Supplementary Fig. S17a) or overlapped with LEs/LYs in COS-7 cells (Supplementary Fig. S17b). Finally, being vacuolar structures, their colocalization with aquaporin proteins were examined, which are channels that facilitate water transport across the plasma membrane and the endosomal membrane^40^. As representative data, we found that aquaporin-2-EGFP (AQP-2-EGFP) colocalized with the LE/LY compartments, but exhibited no overlap with DBs (Fig. 6g). Other aquaporin proteins did not colocalize with DBs (data not shown). Therefore, our data suggest that DBs represent organelles with molecular profiles distinct from endosomal compartments, despite the presence of some partially shared proteins and lipids.

## Discussion

Because information from multiple raw images is merged to reconstruct one frame in ODT microscopy, movements of any structures in live cells may cause motion blur and compromise resolution, as occurs in SIM reconstruction^14^. For example, the movement of an LE/LY across a distance larger than the spatial resolution of our system (~200 nm) could lead to motion blur and reduced image contrast, which required the acquisition time needed for one reconstructed frame to be <1.38 s (> 95% LE/LY movement, Supplementary Note 1.1). Increased spatial resolution or prolonged exposure would lead to the distribution of the LE/LY signal across an even larger field of view and the ultimate disappearance of the structure into the background noise. Similarly, because ER tubules and junctions also undergo rapid motions in live cells^41^, they have never been observed by any ODT microscopes in live cells previously, and such an observation has even been deemed to be impossible^10^. Therefore, the spatial resolution must be matched with the corresponding temporal resolution to enable the maximal resolution achievable in live-cell ODT imaging, which has been overlooked in previous designs^6,9,13^. To maintain contrast and resolution, despite limited light illumination, it is crucial for fast ODT microscopes to have sufficiently high sensitivity. In addition to the careful design and alignment of the optic path, we used an sCMOS camera with a large full-well electron capacity and mechanical galvo-scanning mirrors with reduced optical distortion compared with the digital micromirror device. The subsequent wobbling of the illumination angle and the misaligned stitching of the Ewald sphere in ODT during high-speed mechanical scanning in long-term live-cell imaging^42^ were resolved by the VISA algorithm, which precisely determines the scanning vectors of the angle-varying illumination and minimizes the stitching error. These factors all contributed to the presence of sufficient photon flux during short exposures (Supplementary Note 1) and superior performance of our ODT microscopy compared with previous attempts.

Compared with fluorescence microscopy, our fast ODT microscopy has several unique advantages. ODT microscopy can be used to image cells, structures, and processes that are susceptible to phototoxicity, such as cell mitosis (Fig. 2). On the other hand, SR fluorescence imaging generated phototoxicity that arrested the COS-7 cell at the late prophase (Supplementary Fig. S11, Supplementary Video 9), which is consistent with the significant phototoxicity of fluorescence microscopy observed upon in vivo imaging of H2B-EGFP during *Caenorhabditis elegans* embryo development^43^. In addition, ODT microscopy could easily detect nonspecific effects due to exogenous protein overexpression. For example, compared with cells loaded with LysoView 488 alone, the sizes of LE/LY structures observed by ODT microscopy in LAMP1-EGFP-overexpressing cells were significantly larger, while those in Rab7-EGFP-overexpressing cells were significantly smaller (Supplementary Fig. S18). Recently, the emergence of membraneless organelles and phase separation between solid and liquid states within live cells were found to be universal mechanisms involved in the mediation of important biological processes^44^. Compared with fluorescence microscopy, which only highlights specific proteins or organelles, ODT microscopy is able to image changes in cellular masses due to phase separation processes because in this method, signal intensity is correlated with the spatial distribution of the material density within the cell^9,23,24^. In fact, we could clearly visualize the condensation of chromatin and the emergence of the nuclear membrane during mitosis in live cells (Fig. 2), which resembled phase separation processes. Finally, ODT can provide a comprehensive map of the organelle interactome, as the total number of organelles that can be detected by ODT in 3D, such as mitochondria, LDs, and LEs/LYs, outnumbers the total number that can be detected by 2D microscopes in only one Z plane (Supplementary Fig. S10). Furthermore, the same cell can also be imaged for an unlimited period of time, which will provide continuous information on long-lasting cellular processes and enable the visualization of rare structures and intermediates.

On the other hand, fluorescence Hessian SIM is also essential. With increased resolution and contrast, Hessian SIM provided more exquisite detail, including mitochondrial cristae and their dynamics in live cells. Further enhancement of the capability of SR-FACT in multicolor fluorescence SR imaging is needed to visualize organelles that currently remain invisible to ODT microscopy, such as the Golgi and peroxisomes. Moreover, with specific labeling, fluorescence SR imaging can also highlight critical proteins/lipids/molecules in spatiotemporal moments of structural and dynamic changes. Finally, by imaging fluorescently labeled probes, SR-FACT allows functional dynamics involving Ca^2+^, voltage, and cAMP to be incorporated into the cellular landscape. However, the use of a low-phototoxicity SR fluorescence microscope with an ODT microscope is nontrivial. Volumetric SR fluorescence SIM requires intense illumination excitation, which causes extensive photobleaching and phototoxicity, and the temporal resolution of this method is limited by the speed of mechanical changes in axial focal planes. Both disadvantages are incompatible with ODT and render live-cell correlative SR imaging in 3D impossible (Supplementary Note 1). Therefore, we used Hessian 2D-SIM, which has been proven to reduce the photon dose by tenfold compared with conventional 2D-SIM^14^, to help in the identification and interpretation of structures seen by the ODT module.

DBs exhibited characteristics distinct from those of known organelles. First, although DBs partially shared some endosomal markers with conventional endosomes, their vesicular lumens had a neutral pH and were mostly enriched with liquid. Both characteristics were different from those of endosomes. Next, hour-long high-resolution ODT imaging revealed the biogenesis of DBs in perinuclear regions enriched with organelles and biomaterials before the final collapse into the plasma membrane, which is also distinct from that of endosomal compartments, which mainly use the opposite endocytic trafficking route. Finally, we showed that water transport proteins, including AQP-2, which mostly resides on endosomes, were mostly absent in DBs, which again suggests their unique identity. Alternatively, if DBs were observed under fluorescence microscopy or label-free microscopy alone, they would be regarded as endocytic intermediates, endosomes, or vacuoles similar to those in plants and yeasts. Only by combining the molecular and landscape information obtained from ODT microscopy and Hessian SIM, we could conclude that DBs represent previously unappreciated organelles, thus highlighting the power of SR-FACT in identifying novel structures.

By intimately interacting with other organelles, including mitochondria and nuclear membranes (Fig. 5f–j), DBs may be an important organizer of the organelle interactome. In particular, we show that different organelles can sequentially interact with one DB to form a multiorganelle complex, in which the DB serves as the hub (Fig. 5k–n). Given that organelle contacts usually involve specific lipid and protein tethers at the interfaces, our data suggest that distinct domains of lipids and proteins may exist in the same DB. Therefore, DBs may facilitate the exchange of material and information among different organelles, some of which are possibly ultimately transported to the plasma membrane. Interestingly, different types of ageing stem cells, which are known to be affected by alterations in the nuclear membrane^36,37^, exhibited a phenotype-associated increase in the number of DBs in hMSCs. These data suggest that nuclear information can be transmitted to DBs either directly or indirectly via DE–nuclear membrane contacts. Large vacuoles (2–3 μm in diameter) are occasionally observed in mammalian cells under abnormal conditions, such as nutrient deprivation, chemical exposure, bacterial toxin treatment, or the inhibition of PI5 kinase^45–52^. These vacuoles may represent a minor population of DBs in normal COS-7 cells that increase in size under pathological stress conditions, which is also consistent with the importance of normal DB trafficking in the maintenance of cellular functions.

In summary, SR-FACT represents a tool that provides a holistic view of the organelle interactome in 3D in live cells and highlights the specific organelles/molecules/signaling pathways involved. Due to the dual-mode correlated SR imaging capability, SR-FACT can reveal phenomena that cannot be appreciated by using either one of the imaging modalities alone and often leads to unexpected observations of well-studied processes. With minimal phototoxicity and a lack of special requirements for labeling methods, it also represents a new generation of user-friendly SR microscopy that may generate terabytes of structural and dynamic information and be instrumental in expanding the understanding of cell biological processes in live cells.

## Materials and methods

### Cell maintenance and preparation

COS-7 cells were cultured in high-glucose DMEM (GIBCO, 21063029) supplemented with 10% fetal bovine serum (FBS) (GIBCO) and 1% 100 mM sodium pyruvate solution (Sigma-Aldrich, S8636) in an incubator at 37 °C with 5% CO_2_ until ~75% confluency was reached. HUVECs were isolated and cultured in the M199 medium (Thermo Fisher Scientific, 31100035) supplemented with fibroblast growth factor, heparin, and 20% FBS (GIBCO) or in the ECM medium containing endothelial cell growth supplement (ECGS) and 10% FBS (GIBCO) in an incubator at 37 °C with 5% CO_2_ until ~75% confluency was reached. INS-1 cells were cultured in the RPMI 1640 medium (GIBCO, 11835-030) supplemented with 10% FBS (GIBCO), 1% 100 mM sodium pyruvate solution, and 0.1% 55 mM 2-mercaptoethanol (GIBCO, 21985023) in an incubator at 37 °C with 5% CO_2_ until ~75% confluency was reached. Human fibroblast cells were cultured in high-glucose DMEM (GIBCO, 21063029) supplemented with 20% FBS (GIBCO) in an incubator at 37 °C with 5% CO_2_ until ~75% confluency was reached. All hMSCs were cultured in the hMSC culture medium containing 90% α-MEM + Glutamax (Gibco), 10% FBS (Gemcell, A77E01F), 1% penicillin/streptomycin (Gibco), and 1 ng/mL FGF2 (Joint Protein Central). Dorsal root ganglion (DRG) neurons were isolated from P10 rats. Isolated DRGs were removed from the excess roots and digested in dispase II (Roche, 10888700)/collagenase type II (Worthington Biochemical, LS004176) at 37 °C for 30 min and then centrifuged for another 35 min at room temperature. DRG neuronal cell bodies were seeded onto coverslips coated with 30 µg/ml poly-L-ornithine (Sigma, RNBG3346) and 5 µg/ml laminin (Roche, 11243217001) and cultured in neurobasal medium (GIBCO, 21103049) supplemented with 2% B-27 supplement (GIBCO, A3582801), 2 mM glutamine MAX (GIBCO, 35050061), and 1% penicillin/streptomycin (GIBCO, 15140122) in an incubator at 37 °C with 5% CO_2_. After 48 h of culture in vitro, the DRG neurons were ready for imaging. For the SR-FACT imaging experiments, cells were seeded onto coverslips (Thorlabs, CG15XH).

To label mitochondria, COS-7 cells were incubated with 250 nM MitoTracker™ Green FM (Thermo Fisher Scientific, M7514) in HBSS containing Ca^2+^ and Mg^2+^ but no phenol red (Thermo Fisher Scientific, 14025076) at 37 °C for 15 min before being washed and imaged. To label LDs, COS-7 cells were incubated with 1 × LipidSpot^TM^ 488 (Biotium, 70065-T) in complete cell culture medium at 37 °C for 30 min protected from light before being washed and imaged. To label LEs/LYs, COS-7 cells were incubated with 1 × LysoView^TM^ 488 (Biotium, 70067-T) in complete cell culture medium at 37 °C for 15–30 min protected from light without washing and were then imaged. For the starvation experiments, COS-7 cells were incubated in complete medium or Hanks’ balanced salt solution (HBSS, Gibco 14025) for 18 h prior to imaging.

Cos-7 cells were transfected with LifeAct-EGFP/KDEL-EGFP/Lamin A-EGFP/H2B-EGFP/LAMP1-EGFP/β1,4-galactosyltransferase 1 (B4GALT1)-EGFP/Pex11a-EGFP/LC3-EGFP/Rab7-EGFP/Rab5a-EGFP/Rab9a-EGFP/FYVE-EGFP/EEA1-EGFP/AQP-2-EGFP. Transfections were performed using Lipofectamine^TM^ 2000 (Thermo Fisher Scientific, 11668019) according to the manufacturer’s instructions. Cells were imaged 24–36 h after transfection in a stage-top incubator (TOKAI HIT, INU-ON1-F1).

### Coverslip preparation

To clean the coverslips prior to live-cell imaging, we immersed the coverslips in 10% powdered precision cleaner (Alconox, 1104-1) and sonicated the coverslips for 20 min. After rinsing in deionized water, the coverslips were sonicated in acetone for 15 min, and then sonicated again in 1 M NaOH or KOH for 20 min. Finally, we rinsed the coverslips with deionized water, followed by sonication three times for at least 5 min each time. The washed coverslips were stored in 95–100% ethanol at 4 °C.

### Imaging data analysis and statistics

ImageJ (Fiji) was used to analyse the images. To analyse the DBs (Supplementary Fig. S15), we applied thresholds to individual ODT image stacks for segmentation and calculated the densities of DBs in the Z planes of individual cells with nuclear membrane structures that were clearly visible. For the analysis of other organelles (Supplementary Fig. S10), we manually annotated the ODT data set and segmented the LDs, LEs/LYs, and mitochondria. We calculated the areas of the LDs, LEs/LYs, and mitochondria within an axial volume of 0.86 μm (10 Z planes, with the layer containing the maximal number of organelles serving as the center) to match one Z plane for 2D-SIM and calculated the percentages with respect to the total areas of the LDs, LYs, and mitochondria within the whole cell. We manually tracked the movement of LYs (Supplementary Fig. S1) and DBs (Fig. 5) using the ImageJ plugin TrackMate. MATLAB (Mathworks), OriginPro (OriginLab), Igor Pro (Wavemetrics), and Illustrator (Adobe) were used to analyse the data and to prepare the final images. The average results are shown as the mean ± SEM of the number of experiments indicated. The Mann–Whitney rank-sum test was used to evaluate the statistical significance (*, **, and *** denote *p*-values < 0.05, 0.01, and 0.001, respectively).

## Supplementary information

Supplementary Notes, Figures and Table

Supplementary Video 1

Supplementary Video 2

Supplementary Video 3

Supplementary Video 4

Supplementary Video 5

Supplementary Video 6

Supplementary Video 7

Supplementary Video 8

Supplementary Video 9

Supplementary Video 10

Supplementary Video 11

Supplementary Video 12

Supplementary Video 13

Supplementary Video 14

Supplementary Video 15

Supplementary Video 16

Supplementary Video 17

Supplementary Video 18

Supplementary Video 19

Supplementary Video 20

Supplementary Video 21

Supplementary Video 22

Supplementary Video 23

Supplementary Video 24

Supplementary Video 25

Supplementary Video 26

Supplementary Video 27

Supplementary Video 28

Supplementary Video 29

Supplementary Video 30

Supplementary Video 31

## Data Availability

All data are available in the main text or the supplementary materials.
