# Supplementary material for: Super-resolution fluorescence-assisted diffraction computational tomography reveals the three-dimensional landscape of the cellular organelle interactome
Source: Light Sci Appl. 2020 Jan 28;9:11. doi: 10.1038/s41377-020-0249-4 (PMC6987131; doi:10.1038/s41377-020-0249-4)
Supplement: Supplementary file 1 — Supplementary Notes, Figures and Table [file 41377_2020_249_MOESM1_ESM.pdf]

Supplementary Information for Super-resolution Fluorescence-assisted Diffraction Computational Tomography Reveals the Three-dimensional Landscape of the Cellular Organelle Interactome

Dashan Dong[1,5,12], Xiaoshuai Huang[2,12], Liuju Li[2,12], Heng Mao[3], Yanquan Mo[2], Guangyi Zhang[3], Zhe Zhang[3], Jiayu Shen[4], Wei Liu[1,5], Zeming Wu[7,9], Guanghui Liu[7,8,9], Yanmei Liu[2,10], Hong Yang[1,5,6], Qihuang Gong[1,5,6], Kebin Shi[1,5,6,*], Liangyi Chen[2,11,*]

[1] State Key Laboratory for Mesoscopic Physics and Frontiers Science Center for Nano-optoelectronics, School of Physics, Peking University, Beijing, 100871, China

[2] State Key Laboratory of Membrane Biology, Beijing Key Laboratory of Cardiometabolic Molecular Medicine, Institute of Molecular Medicine, Peking University, Beijing 100871, China

[3] School of Mathematical Sciences, Peking University, Beijing 100871, China

[4] School of Software and Microelectronics, Peking University, Beijing 100871, China

[5] Collaborative Innovation Center of Extreme Optics, Shanxi University, Taiyuan, Shanxi, 030006, China

[6] Collaborative Innovation Center of Quantum Matter, Peking University, Beijing, 100871, China

[7] State Key Laboratory of Stem Cell and Reproductive Biology, Institute of Zoology, Chinese Academy of Sciences, Beijing 100101, China

[8] National Laboratory of Biomacromolecules, Chinese Academy of Sciences Center for Excellence in Biomacromolecules, Institute of Biophysics, Beijing 100101, China.

[9] University of Chinese Academy of Sciences, Beijing 100049, China

[10] Institute for Brain Research and Rehabilitation (IBRR), Guangdong Key Laboratory of Mental Health and Cognitive Science, South China Normal University, Guangzhou, China

[11] PKU-IDG/McGovern Institute for Brain Research, Beijing 100871, China

[12] D. D., X. H. and L. L. contributed equally to this work.

[*] lychen@pku.edu.cn, kebinshi@pku.edu.cn

Supplementary Notes

1. Design principles of SR-FACT

The principles underlying the development of SR-FACT works in two major aspects. For the ODT microscope, we design the new algorithm and the hardware to simultaneously enable maximal imaging speed to capture dynamics in live cells and to maintain sufficient photon flux for maximal sensitivity. For dual-mode correlated imaging, we used SR fluorescence microscopy to guide the interpretation of structures observed by the ODT microscope along with minimal phototoxicity and fast sampling speed. In addition, the ODT module can also provide contextual information for proteins highlighted by the fluorescence microscope. The details are listed below:

1.1 Regarding the ODT system

Because information from multiple raw images is merged to reconstruct one volumetric image in ODT microscopy, movements of structures in live cells may cause motion artifacts and compromise resolution, as shown previously for SIM reconstruction[1]. For example, LYs moved at an average speed of 43 nm/s in live COS-7 cells as deduced from their manually tracked trajectories, whereas ~95% of the LYs moved below a speed of 145 nm/s (**Figure S1**). If an LY moved across a distance larger than the spatial resolution of the system within the acquisition time needed for one reconstructed frame, it would lead to motion artifacts from reduced contrast and even the disappearance of the LY in the final ODT images. Therefore, higher theoretical resolution adversely compromised the performance of the system during live cell imaging, and spatial resolution must be matched with corresponding temporal resolution to enable maximal resolution achievable in live-cell ODT imaging. All previous ODT microscopes fell short on temporal resolution, which readily explained the failure of these microscopes in the detection of LYs in live cells despite high theoretical spatial resolution (**Figure S1**)[2-5]. Similarly, tubular ER structures have never been observed by any ODT microscopes in live cells previously, and such an observation has even been deemed impossible[6], which may be due to the same reason, that is, ER tubules and junctions undergo rapid motions in live cells[7].

With the increased imaging speed, it is important for ODT microscopes to have sufficiently high sensitivity within shorter exposure time. The phase and amplitude sensitivity in interferometric

microscopy is fundamentally determined by the total photon flux during imaging[8]. Thus, the maximal sensitivity of ODT microscopy is determined by the total photon flux during imaging and equals the number of images multiplied by the maximal photon capacity of the camera. Even at a total imaging time of 1.22 s, our system provided sufficient total photon flux, next only to the systems in (Kim et al., 2016)[4] and (Simon et al., 2017)[9], both of which were much slower than our system with imaging time of 3~4 seconds.

To achieve high temporal resolution, we could build the tomographic illumination system based on either fasting scanning galvo-mirrors[10] or non-scanning digital micro-mirror device (DMD)[5]. The protection window of DMD may lead to coherent noise in ODT images and decrease the signal to noise ratio[11], and the optical surface flatness of the DMD is inferior to galvo mirrors, which induces additional illumination aberration[12]. Therefore, we chose galvo-mirrors and drove them to their speed limit. As a result, mechanical wobbling and instability led to deviation of the illumination wave vectors from the designated angles during long-term high-speed scanning of illumination (**Figure S5**). This effect significantly compromised both the contrast and resolution of the reconstructed images[13]. We have developed the VISA algorithm, which is discussed in details in **Supplementary Note 5**. In summary, our ODT microscope is capable of achieving its theoretical spatial resolution and obtaining images of similar or even better quality within shorter exposure intervals than previous systems.

1.2 Regarding correlative imaging

For correlative imaging, simply merging 3D SR fluorescence microscopy with ODT microscopy significantly compromised the performance of the whole system. For example, while the minimal photobleaching and phototoxicity characteristics endow the ODT system with long-term live-cell imaging capability, volumetric SR fluorescence SIM requires intense illumination excitation, which causes extensive photobleaching and phototoxicity. In addition, the slow speed of the mechanical movement of the axial focal plane of the objective to obtain volumetric reconstruction in any SR fluorescence microscopy methods is also incompatible with the rapid volumetric imaging capacity of ODT.

We designed SR-FACT to achieve synchronized dual-mode (ODT and SIM) long-term imaging in live cells. In contrast to providing complete complementary information to ODT, we used SR fluorescence microscopy to guide the interpretation of structures observed by the ODT module. Therefore, we applied Hessian 2D-SIM, which has been proven to reduce the photon dose by ten-fold compared to conventional 2D-SIM[1]. The long-term imaging capacity of SR-FACT was demonstrated by the one and half hour imaging of H2B-EGFP during cell division (**Figure S11**).

2. Hardware configuration for SR-FACT imaging

2.1 Optics

Our SR-FACT system was built on a commercial inverted microscope (IX73, Olympus) equipped with a high numerical aperture (NA) objective (OBJ2 in **Figure S2,** 100 × 1.45 NA Oil, ApoN, Olympus). In addition to OBJ2, lens L4, the dichroic mirror DM1 (ZT405/488/561/640-phase R, Chroma) and DM2 (DMLP550L, Thorlabs) were shared by both the ODT and Hessian SIM modules. In the ODT module, a single longitudinal-mode 561-nm laser (MSL-FN-561-50mW, Changchun New Industries Optoelectronics Technology) was modulated by an acoustic optical modulator (AOM, CETC Chongqing Acousitc-Optic-Electric Co., LTD) and divided into two paths by a polarization beam splitter (CCM1-PBS251, Thorlabs). One beam was used to illuminate samples, while the other served as the reference for hologram recording. Both beams were coupling into polarization maintaining single mode fibers (PM460-HP, Connet Fiber Optics) with the fiber collimators (PAF2-7A, Thorlabs). The illumination light exiting from the fiber was collimated with a lens (L1, AC254-040-A, Thorlabs) before being relayed onto a pair of one-dimension galvo-mirrors (GM, 2× GVS211/M, Thorlabs) placed on the conjugation planes of a 4-f system (2× AC254-100-A, Thorlabs) that scanned the illumination beam. The scanned beam was then focused by another lens (L2, AC508-180-A, Thorlabs) to enter the back focal plane of a water immersion objective OBJ1 (60× 1.0 NA, LUMPlanFLN, Olympus). The illumination beam that passed through the sample via a tilted angle on the stage was collected by the objective OBJ2, followed by its reflection by the dichroic mirror DM1 and collimation by a tube lens (ITL200, Thorlabs). After passing through another dichroic mirror DM2, the transmitted beam was expanded by a pair of relay lenses (L7 and L9, TTL180-A and AC508-250-A, Thorlabs). Finally, a beam splitter (BS013, Thorlabs) was used to combine the signal beam with the reference beam collimated by the lens L10 (AC508-100, Thorlabs). The off-axis holograms at each

illumination angle were captured by the sCMOS camera (ORCA-Flash 4.0 V3, Hamamatsu).

The optical layout in the Hessian SIM module is similar to that reported in our previous paper (Huang et al., 2018). In short, we used a 488-nm single longitudinal-mode laser (Sapphire 488LP-200, Coherent) as the illumination source and an acoustic optical tunable filter (AOTF, AA Opto-Electronic, France) to switch and adjust the illumination power of the laser. Therefore, the Hessian SIM could generate and switch between excitation patterns created by various diffraction-limited gratings generated by the ferroelectric liquid crystal on silicon SLM (SXGA-3DM, Fourth Dimension Display) with a high frame rate. To maximize the modulation contrast of the excitation pattern, it was necessary to change the polarization of the input beams to S-polarization. We used a Liquid Crystal Variable Retarder (LVR-200-VIS-1L-TSC, Meadowlark) combined with a quarter-wave plate to rotate the polarization of the laser. The ±1 orders of excitation beams passed through a pair of lenses (L5 and L6, AC508-500-A and AC508-300-A, Thorlabs) and penetrated the PR before being reflected by the DM2; the beams were collimated by L4 and reflected by DM1. Finally, the two beams entered the back focal plane of the OBJ2 and interfered at the sample plane. At the emission path, we designed a synchronization paradigm that efficiently coordinated the pattern generation of the SLM and the camera readout interval and used a sCMOS camera with 82% peak quantum efficiency (ORCA-Flash4.0 V2, Hamamatsu) to detect the emission fluorescence passing through the DM1 and filter.

2.2 System synchronization

In ODT imaging, a group of 240 raw holograms at different tilting illumination angles were used to reconstruct one volume image. The first and the last holograms of each group were set as the group marker with the illumination directions perpendicular to the sample plane. At other illumination angles, the galvo-mirrors were controlled to scan the focal points on the back focal plane of the illumination objective lens on a circle along the edge of the entrance pupil. To minimize the exposure times, the movement of galvo-mirrors was synchronized with the exposure of the sCMOS camera. The time sequences for the ODT acquisition followed by SIM acquisition are shown in **Figure S3**. We used the synchronous readout mode of sCMOS camera together with an AOM to control the laser lighting synchronously during exposures of rows. Because every row exposure is synchronized with the laser illumination, this mode is equivalent to the using of a global-shutter mode. To minimize the vibrational noise and time-dependent motion artifacts, we reduced the equivalent exposure time to 50 μs, as shown

by the AOM trigger signal in the lower inset in **Figure S3**. Notably, while the sCMOS camera was working on the synchronous readout mode, we did not use the first hologram of each 240 frame group data in order to avoid variations in actual exposure time and leaked excitation of fluorescence. To eliminate the high-order residue background in phase and homogenize the illumination amplitude, we captured an extra group of background holograms with only the coverslip under the microscope for calibrating. The whole cycle of ODT and SIM acquisitions required 1.49 s, which is sufficiently fast for most of the live cell dynamics studied here, as demonstrated by the colocalization of same organelles captured alternatively by the ODT and SIM modules (**Figure 3**).

3. Rytov based ODT reconstruction

Due to the inhomogeneous distribution of the refractive index (RI) within the sample volume, light passing through biological samples generates a scattered optical field. In diffraction optics, the RI spatial distribution $n(\vec{r})$ can be quantitatively correlated to the scattering potential $f(\vec{r})$ by the equation[14]:

$$f(\vec{r}) = -k_0^2[(n(\vec{r})/n_0)^2 - 1] \tag{1}$$

where $\vec{r}$ denotes spatial coordinates, $k_0 = 2\pi n_0/\lambda$ is the averaged wave vector of light in the medium surrounding the sample, n_0 is the refractive index of the medium, and λ is the wavelength of the incident laser. The scattering process can be described by the Helmholtz equation:

$$\nabla^2 U_\mathrm{s}(\vec{r}) + k_0^2 U_\mathrm{s}(\vec{r}) = f(\vec{r}) U_\mathrm{total}(\vec{r}) \tag{2}$$

where $U_\mathrm{total}(\vec{r})$ is the total electric field of the light after it passes through the sample, and $U_\mathrm{s}(\vec{r})$ is the electric field of the scattered light signal induced by the inhomogeneous RI distribution in the sample. If the scattered optical field is much smaller than the incident laser fields, as experienced in light passing through thin samples, we can use the Born approximation in solving equation (2). Under the first-order Born approximation, the scattering potential is correlated with the scattering field by[15]:

$$\hat{F}\left(K_x^j, K_y^j, K_z^j\right) = 2ik_z \hat{U}_\mathrm{S-Born}^j\left(k_x, k_y\right) \tag{3}$$

where

$$K_x^j = k_x - k_{x0}^j,\ K_y^j = k_y - k_{y0}^j,\ K_z^j = k_z - k_{z0}^j \tag{4}$$

Here, $\hat{F}\left(K_x^j, K_y^j, K_z^j\right)$ is the 3D Fourier transform of the scattering potential $f(\vec{r})$, $K_x^j, K_y^j\ and\ K_z^j$

denote the spatial frequency components of the scattering potential, k_x , k_y and $k_z = \sqrt{k_0^2 - k_x^2 - k_y^2}$ are the wave vectors of the measured scattered optical field; $\vec{k}_0^j(k_{x0}^j, k_{y0}^j, k_{z0}^j)$ denotes the wave vectors of the incident laser field under the jth illumination angle $(j = 0, 1, \ldots, l)$, and $\hat{U}_{\text{S-Born}}^j(k_x, k_y)$ is the Fourier transform of the measured 2D scattered optical field under the jth illumination angle based on the first-order Born approximation.

The first-order Born approximation is only valid when the scattered optical field is much smaller than the incident field. Considering the transparent nature of biological cells, the scattering confers the major source of the phase change. While a live cell usually occupies ~10 μm in the axial direction and contains different organelles with a certain volume of constant RI distributions, the Born approximation failed to account for the phase accumulation across the optical path passing through a cell. Under such circumstances, the Rytov approximation is a more accurate model[10].

Under the Rytov approximation[16], a complex Rytov field $U_{\text{Rytov}}^j(x, y)$ is used to describe the scattering as the summation of all phase perturbations to the incident optical field. The scattered total field can be written as:

$$U_{\text{total}}^j(x, y) = U_{\text{in}}^j(x, y) e^{U_{\text{Rytov}}^j(x,y)} \quad (5)$$

where $U_{\text{in}}^j(x, y) = U_0 e^{ik_{0,x}^j x + k_{0,y}^j y}$ is the jth imaged incident field, U_0 denotes the amplitude of the imaged incident field, $U_{\text{total}}^j(x, y)$ is the transmitted total field recorded by the experiment. By substituting the Born term in equation (3), the 3D Fourier transform of the scattering potential under the 2D Rytov approximation can be derived as[10]

$$\hat{F}(K_x^j, K_y^j, K_z^j) = 2ik_z \hat{U}_{\text{Rytov}}^j(k_x - k_{x0}^j, k_y - k_{y0}^j) \quad (6)$$

where $\hat{F}$ is the 3D Fourier transform of the scattering potential, and $\hat{U}_{\text{Rytov}}^j$ is the Fourier transform of the measured Rytov field under the jth illumination.

We recorded the off-axis hologram to obtain the 2D complex optical fields of $U_{\text{total}}^j(x, y)$ under different illumination angles (discussed in **Supplementary Note, Part 4**). $U_{\text{in}}^j(x, y)$ is the background optical field obtained under the same detection configuration in the absence of samples. Based on equation (5), the Rytov complex field can be calculated as:

$$U_{\mathrm{Rytov}}^{j}(x,y)=\ln\frac{U_{\mathrm{total}}^{j}}{U_{\mathrm{in}}^{j}}=\ln\frac{\left|U_{\mathrm{total}}^{j}\right|}{\left|U_{\mathrm{in}}^{j}\right|+\delta}+\mathrm{i}\cdot\mathrm{unwrap}\left[\mathrm{ang}\left(U_{\mathrm{total}}^{j}\right)-\mathrm{ang}\left(U_{\mathrm{in}}^{j}\right)\right] \tag{7}$$

where δ is a regularization constant to suppress noise, and the ang() operation denotes the calculation of the 2D phase distribution for U_{total}^{j} and U_{in}^{j}. In equation (7), $\mathrm{ang}\left(U_{\mathrm{total}}^{j}\right)-\mathrm{ang}\left(U_{\mathrm{in}}^{j}\right)$ is modulo 2π, i.e., continuous phase information is sampled in a discrete wrapped phase ranging from $0-2\pi$. As a result, 2D phase unwrapping is required under the 2D Rytov approximation, which essentially add or minus the correct integral 2π to compensate for the phase discontinuity of the detected wave front.

In experiments, given the low-pass filtering nature of the imaging system, the measured spatial frequency components of the scattering potential $\hat{F}_{\mathrm{measure}}\left(K_x^j,K_y^j,K_z^j\right)$ under *j*-th illumination angle are essentially the actual scattering potential $\hat{F}_{\mathrm{real}}\left(K_x^j,K_y^j,K_z^j\right)$ filtered by the complex coherent transfer function (CTF) $C\left(k_x,k_y,k_z\right)$ (measured in **Figure S8**) as expressed by

$$\hat{F}_{\mathrm{measure}}\left(K_x^j,K_y^j,K_z^j\right)=\hat{F}_{\mathrm{real}}\left(K_x^j,K_y^j,K_z^j\right)C\left(k_x,k_y,k_z\right) \tag{8}$$

Therefore, the 3D spatial frequency relation of the scattering potential shown in equation (5) can be revised as

$$\hat{F}\left(K_x^j,K_y^j,K_z^j\right)=\frac{2ik_z\hat{U}_{\mathrm{Rytov}}^{j}\left(k_x-k_{x0}^{j},k_y-k_{y0}^{j}\right)}{C\left(k_x,k_y,k_z\right)} \tag{9}$$

Equation (9) indicates the retrieval of the 3D scattering potential under the jth illumination. In ODT imaging, a group of tomographic images are recorded under different illumination angles, and related Rytov field distributions can be obtained by using equation (7). Thereafter, we stitched the measured Rytov field distributions obtained at different illumination angles into the spatial frequency domain to obtain the total scattering potential shown in equation (9). To avoid repeatedly counting overlapped frequency components during the frequency spectrum stitching, we added a Wiener filtering procedure before integrating all illumination angles to obtain the total scattering potential, as shown in equation (10)

$$f(\vec{r})=IFFT.\left\{\frac{\sum_j 2i\left(K_z^j+k_{z0}^j\right)\hat{U}_{\mathrm{Rytov}}^{j}\left(K_x^j,K_y^j,K_z^j\right)C\left(K_x^j+k_{x0}^j,K_y^j+k_{y0}^j,K_z^j+k_{z0}^j\right)}{\sum_j\left|C\left(K_x^j+k_{y0}^j,K_y^j+k_{y0}^j,K_z^j+k_{z0}^j\right)\right|^2+\alpha^2}\right\} \tag{10}$$

where $\hat{U}_{\mathrm{Rytov}}^{j}\left(K_x^j,K_y^j,K_z^j\right)$ is the measured 2D $\hat{U}_{Rytov}^{j}$ field recasted in the 3D spatial frequency

domain as described by equation (4), and $C\left(K_x^j + k_{x0}^j, K_y^j + k_{y0}^j, K_z^j + k_{z0}^j\right)$ is the recasted CTF in the 3D spatial frequency domain with a 3D wave vector shifting by $\vec{k}_0^j$ under the jth illumination. α is the Wiener filtering factor[17] used to reduce computational artifacts.

4. Principles of off-axis hologram

In an off-axis holographic recording, we use a known optical plane wave as denoted by $Re^{i(k_{ref,x}x + k_{ref,y}y)}$ as the reference, which interferes with the optical signal field U. Given that R is the amplitude of the reference field, U is the complex signal optical fields to be measured, and k_{ref} is the 2D wave vectors of the reference beam, the detected intensity $I(x, y)$ of the hologram recorded on the camera can be written as

$$I(x, y) = |U|^2 + |R|^2 + R^* U e^{-i(k_{\mathrm{ref},x}x + k_{\mathrm{ref},y}y)} + c.c. \tag{11}$$

Where $c.c.$ denotes complex conjugation of the third term in right side of equation (11). By performing 2D Fourier transform of $I(x, y)$ and subsequently carrying spatial spectrum filtering centered at $(k_{\mathrm{ref},x}, k_{\mathrm{ref,y}})$ as indicated by the third term in right side of equation (11), the complex signal field U can be retrieved by performing inverse Fourier transform on the spectrally filtered component with the prior knowledge of a known plane wave reference field.

5. Algorithm implementation

Following the principle described above, the ODT reconstruction algorithm is schematically shown in **Figure S4**. Initially, l holograms ($l = 240$ used in the current setting) was taken as background with a coverslip without cells attached. During time-lapse live cell imaging, l holograms with tomographic illumination angles identical to the background data acquisition were recorded at each temporal point.

Step 1 Holographic processing

As shown in **Figure S4**, the amplitude and phase of both background and time-lapse data sets were first extracted by the standard off-axis holographic processing procedure as explained in **Section 3**, which consists of the Fourier transform of raw images, spectrum filtering of the Fourier domain, and inverse Fourier transform. In addition, measuring the distance between the DC term and the brightest point of the modulation term in the Fourier domain yielded interference angles between the signal and

the reference beams, termed as the transverse wave vector k_{ref}^{j} (in the unit of pixel). Because of the circular scanning scheme, the reference wave vector is calculated as the mean of l detected angles because scanning illumination wave vectors are canceled out during the averaging process,

$$k_{\mathrm{ref}} = \frac{\sum_{j=0}^{l} k_{\mathrm{ref}}^{j}}{l} \quad (12)$$

Therefore, an approximation of the illumination wave vector for the j_{th} hologram in the total l holograms can be given by

$$k_0^j = k_{\mathrm{ref}}^{j} - k_{\mathrm{ref}} \quad (13)$$

Step 2 Precise determination of the scanning wave vector

One problem with long-term high-speed scanning of illumination is that mechanical wobbling and instability of the galvo-mirrors may lead to deviation of the illumination wave vectors from the designated angles. As shown in **Figure S5**, the measured illumination wave vectors at 100 time points significantly deviated from those of reference images obtained in the absence of a sample. The standard deviations of k_x and k_y were 0.0061 μm^{-1} and 0.008 μm^{-1}, respectively, corresponding to ~0.05 degree error in the illumination wave vector. Such errors in illumination wave vectors will induce misalignment in 3D spatial frequency spectrum stitching and consequently degrade the imaging quality, as determined by equations (10) and (11) and discussed in the reference[13].

To address this problem, we proposed the VISA algorithm that precisely determined the actual illumination wave vector and minimized the residue slope of the unwrapped phase distribution extracted from holographic recordings. The pipeline of the VISA, which consisted of four major steps, is shown in **Figure S6**. First, we multiplied the phase image extracted from holograms by the digital phase shifting term $e^{ik_{0,}^{j}r}$, which provided an initial crude estimation of the 2D illumination vector k_0^j based on equation (13). Next, the frequency shifted phase image was unwrapped using the least-square method proposed previously[18]. By linearly fitting the residue slope α^j in the k_x and k_y directions in the unwrapped image, an updated wave vector was generated by adding the feedback term as given below:

$$k_{0,\mathrm{new}}^{j} = k_{0,\mathrm{old}}^{j} - \frac{\alpha^j}{2\pi} \quad (14)$$

The updated wave vector is then used as the input for the next round of iteration until the residue

slope α^j reaches the preset threshold (0.00001 for $\alpha^j/2\pi$ in our experiments). After three iterations, the residue slope was reduced to an extent less than the threshold. While the RI distributions within the COS-7 cell were significantly distorted in the absence of VISA processing, the VISA-corrected image was more homogenous and exhibited better contrast (**Figure S7**). In fact, ER tubular structures that were previously concluded to be not visible using the ODT microscope[6] were clearly resolvable after VISA correction (**Figure 3a** and **Figure S7**).

Having accurately determined the illumination wave vectors of the sample at individual time points, we calculated their differences with the wave vectors extracted from the background hologram. During the recording of a full set of holograms, there were sometimes missing frames due to failed data logging to the hard drive. Therefore, we also included a step to identify the missing frames, followed by removing missing frames and regroup the raw data for hologram reconstruction.

Steps 3, 4 and 5 Compute the Rytov phase, perform spatial spectrum stitching and determine scattering potential

The amplitude and unwrapped phase from different time points were normalized by those of the background hologram. The Rytov phase component could be calculated according to equation (7) (see also Step 3 in **Figure S4**), which was then filtered and stitched to the 3D spatial spectrum using equation (10) (Step 4 in **Figure S4**). After performing Wiener filtering described in equation (10), the 3D distribution of RIs was reconstructed using equation (1) (Step 5 in **Figure S4**).

We used multithread programing with a C++ compiler to enable offline data processing on a high-performance computing platform. The time-lapse holograms and background holograms were feeded into the program as TIFF-stack files. The result was also saved as TIFF-stack files and can be easily analyzed using ImageJ. LibTIFF 4.0.9 and FFTW 3.3.8 library is used for data I/O and efficient FFT computation during image reconstruction.

6. Coherent transfer function (CTF)

In the ODT imaging scenario, the measured scattering complex field $U^j_{\mathrm{measure}}(x,y,z)$ can be expressed as the convolution of the actual signal complex field $U^j_{\mathrm{S}}(x,y,z)$ with the complex point spread function (PSF) $h(x,y)$:

$$U^j_{\mathrm{measure}}(x,y,z) = \iiint U^j_S(x'-x,y'-y,z'-z)h(x',y')G(x',y',z')\,\mathrm{d}x'\mathrm{d}y'\mathrm{d}z' \tag{20}$$

in which $G(x', y', z')$ is the far field propagation term (Born and Wolf, 1980), and it can be simplify as

$$G(\vec{r}) = \frac{1}{4\pi} \cdot \frac{e^{ik_0|\vec{r}|}}{|\vec{r}|} \tag{21}$$

where $\vec{r}$ denotes spatial coordinates (x, y, z). In the spatial frequency domain, equation (20) can be rewritten as

$$\widehat{U}_{\text{measure}}(k_x, k_y, k_z) = \widehat{U}_S^j(k_x, k_y, k_z) C(k_x, k_y, k_z) \tag{22}$$

where $\widehat{U}_{measure}(k_x, k_y, k_z)$ and $\widehat{U}_S^j(k_x, k_y, k_z)$ are the 3D Fourier transforms of $U_{measure}^j$ and U_S^j, respectively, while $C(k_x, k_y, k_z)$ is the CTF. Thus, the CTF can be expressed as

$$C(k_x, k_y, k_z) = F.T.^{3\text{D}}\{h(x', y')G(x', y', z')\} \tag{23}$$

Because the field propagates as a monochromatic wave with a constant wave vector k_0, $C(k_x, k_y, k_z)$ is the contoured projection of 2D CTF $C(k_x, k_y)$ onto a spherical surface determined by k_{0z} in the spatial frequency domain as indicated by equation (23).

Experimentally, we measured the CTF of the system with a nanoscale aperture on an aluminum surface. A nanohole (100 nm in diameter) was fabricated with the focus ion beam[3]. To eliminate the noise introduced by the nonuniform reference beam and weakly transmitted signal, multiple images from different illumination angles were averaged to yield the mean CTF. Following the procedure described in **Supplementary Part 4**, we retrieved both the amplitude and phase of the 2D CTF from the hologram of the nanohole (**Figure S8**), which led to the calculation of the 3D CTF $C(k_x, k_y, k_z)$ with equation (23).

7. Analysis and characterization of resolution in SR-FACT

According to Rayleigh's criterion, the resolution of the coherent imaging system is given by[19]

$$d_{\text{min,coh}} = 0.82 \frac{\lambda}{\text{NA}} \tag{24}$$

where λ is the illumination wavelength and NA is the optical system numerical aperture (NA). In the ODT imaging system, the rotational illumination extends the transverse spatial frequency limit, as demonstrated by the two mirrored incident angles along the maximum NA of illumination in **Figure S9**. In theory, the scattered field in the ODT essentially poses a frequency shift in the detection

frequency domain (equation 3), which extends the bandwidth in the transverse frequency domain by the following equation:

$$k_{\parallel,odt} = k_{\parallel,det} + k_{\parallel,ill} \quad (25)$$

where $k_{\parallel,ill}$ and $k_{\parallel,det}$ indicate the transverse projections of the maximum NA of the illumination objective and detection objective, respectively. The NA of the illumination objective lens was 1.0. Although we used a 1.45 NA objective, the maximal NA detected by the object is still limited by the immerse medium, which has a low RI. Given that these live cells were maintained in PBS ($n \approx 1.333$), the effective NA of the ODT system can be given as

$$NA_{\text{eff}} = NA_{\text{det}} + NA_{\text{ill}} \approx 2.33 \quad (26)$$

Because we used a 561-nm laser for illumination, the theoretical transverse resolution in the ODT system is ~197 nm, which has been proven by the benchmark experiment in the COS-7 cell (**Figure 1d-1f**).

The axial resolution of the ODT system is complicated by the nonuniform longitudinal distribution in the spatial frequency (**Figure S9c**, lower panel). Instead, the longitudinal spatial frequency bandwidth essentially becomes dependent on the transverse frequency. A missing core appears in the $k_{\perp}$ direction near the low $k_{\parallel}$ area, indicating that the actual PSF has a large FWHM in the longitudinal direction. When imaging live cells, especially those with high transverse frequency components in cellular structures, the corresponding bandwidth in the longitudinal direction increases, as shown in **Figure S8c**, where the longitudinal frequency bandwidth can be estimated as

$$k_{\perp,\text{odt}} \approx \frac{2\pi n_0}{\lambda} \cos \text{asin} \frac{NA_{\text{ill}}}{n_0} \quad (27)$$

where $n_0 = 1.33$, and $\lambda = 561$ nm in the experiments, yielding a simply estimated longitude resolution for our system of

$$\Delta z = 0.82 \times \frac{2\pi}{k_{\perp,\text{odt}}} \approx 525\ nm \quad (28)$$

Reference

1. Huang, X. S. *et al*. Fast, long-term, super-resolution imaging with Hessian structured illumination microscopy. *Nature Biotechnology* **36**, 451-459 (2018).
2. Chowdhury, S., Eldridge, W. J., Wax, A. & Izatt, J. Refractive index tomography with structured illumination. *Optica* **4**, 537-545 (2017).
3. Cotte, Y. *et al*. Marker-free phase nanoscopy. *Nature Photonics* **7**, 113-117 (2013).
4. Kim, K. *et al*. Three-dimensional label-free imaging and quantification of lipid droplets in live hepatocytes. *Scientific Rep* **6**, 36815 (2016).
5. Shin, S., Kim, D., Kim, K. & Park, Y. Super-resolution three-dimensional fluorescence and optical diffraction tomography of live cells using structured illumination generated by a digital micromirror device. *Scientific Reports* **8**, 1983 (2018).
6. Sandoz, P.A. *et al*. Label free 3D analysis of organelles in living cells by refractive index shows pre-mitotic organelle spinning in mammalian stem cells. Preprint at https://doi.org/10.1101/407239 (2018).
7. Nixon-Abell, J. *et al*. Increased spatiotemporal resolution reveals highly dynamic dense tubular matrices in the peripheral ER. *Science* **354**, aaf3928 (2016).
8. Hosseini, P. *et al*. Pushing phase and amplitude sensitivity limits in interferometric microscopy. *Optics Letters* **41**, 1656-1659 (2016).
9. Simon, B. *et al*. Tomographic diffractive microscopy with isotropic resolution. *Optica* **4**, 460-463 (2017).
10. Sung, Y. *et al*. Optical diffraction tomography for high resolution live cell imaging. *Optics Express* **17**, 266-277 (2009).
11. Choi, G. *et al*. Cycle-consistent deep learning approach to coherent noise reduction in optical diffraction tomography. *Optics Express* **27**, 4927-4943 (2019).
12. Cox, M. A., Cheng, L. & Forbes, A. Digital micro-mirror devices for laser beam shaping. In Fifth Conference on Sensors, MEMS, and Electro-Optic Systems. Vol. 11043 (2019).
13. Kuś, A. Illumination-related errors in limited-angle optical diffraction tomography. *Applied Optics* **56**, 9247-9256 (2017).
14. Born, M. & Wolf, E. Principles of Optics. (Pergamon, New York, 1980).
15. Wolf, E. Three-dimensional structure determination of semi-transparent objects from holographic data. *Optics Communications* **1**, 153-156 (1969).
16. Devaney, A. J. Inverse-Scattering Theory within the Rytov Approximation. *Optics Letters* **6**, 374-376 (1981).
17. Kailath, T., Sayed, A. H. & Hassibi, B. Linear estimation. (Prentice Hall, 2000).
18. Ghiglia, D. C. & Romero, L. A. Robust two-dimensional weighted and unweighted phase unwrapping that uses fast transforms and iterative methods. *JOSA A* **11**, 107-117 (1994).
19. Den Dekker, A. J. & Van den Bos, A. Resolution: a survey. *JOSA A* **14**, 547-557 (1997).
20. Wu, H., Carvalho, P. & Voeltz, G. K. Here, there, and everywhere: The importance of ER membrane contact sites. *Science* **361**, eaan5835 (2018).
21. Zhang, W. *et al*. Aging stem cells. A Werner syndrome stem cell model unveils heterochromatin alterations as a driver of human aging. *Science* **348**, 1160-1163 (2015).

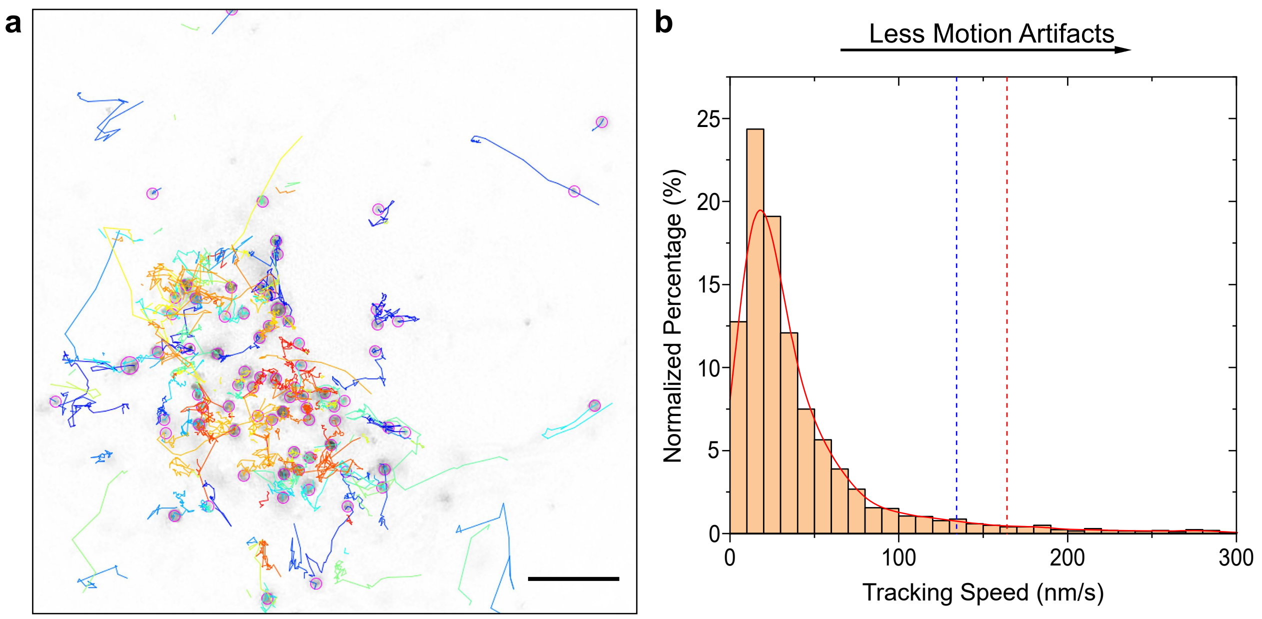

Figure S1 | Analysis of possible motion artifacts of different ODT microscopes in imaging fast moving lysosomes. (**a**)Trajectories of different lysosomes from the time-lapse SIM image are shown (**Movie 4**). Different trajectories were shown with different colors. Scale bar, 5 μm. (**b**)Distribution of moving speeds of 4376 link trajectories in (**a**). Boundaries for the appearance of motion artifacts were determined by spatial resolutions divided by imaging duration. We used red dash line to indicate boundary for our ODT microscope at 200 nm / 1.22 s = 164 nm/s, and blue dash line to indicate boundary for our SR-FACT at 200 nm / (1.22+0.27) s = 134 nm/s.

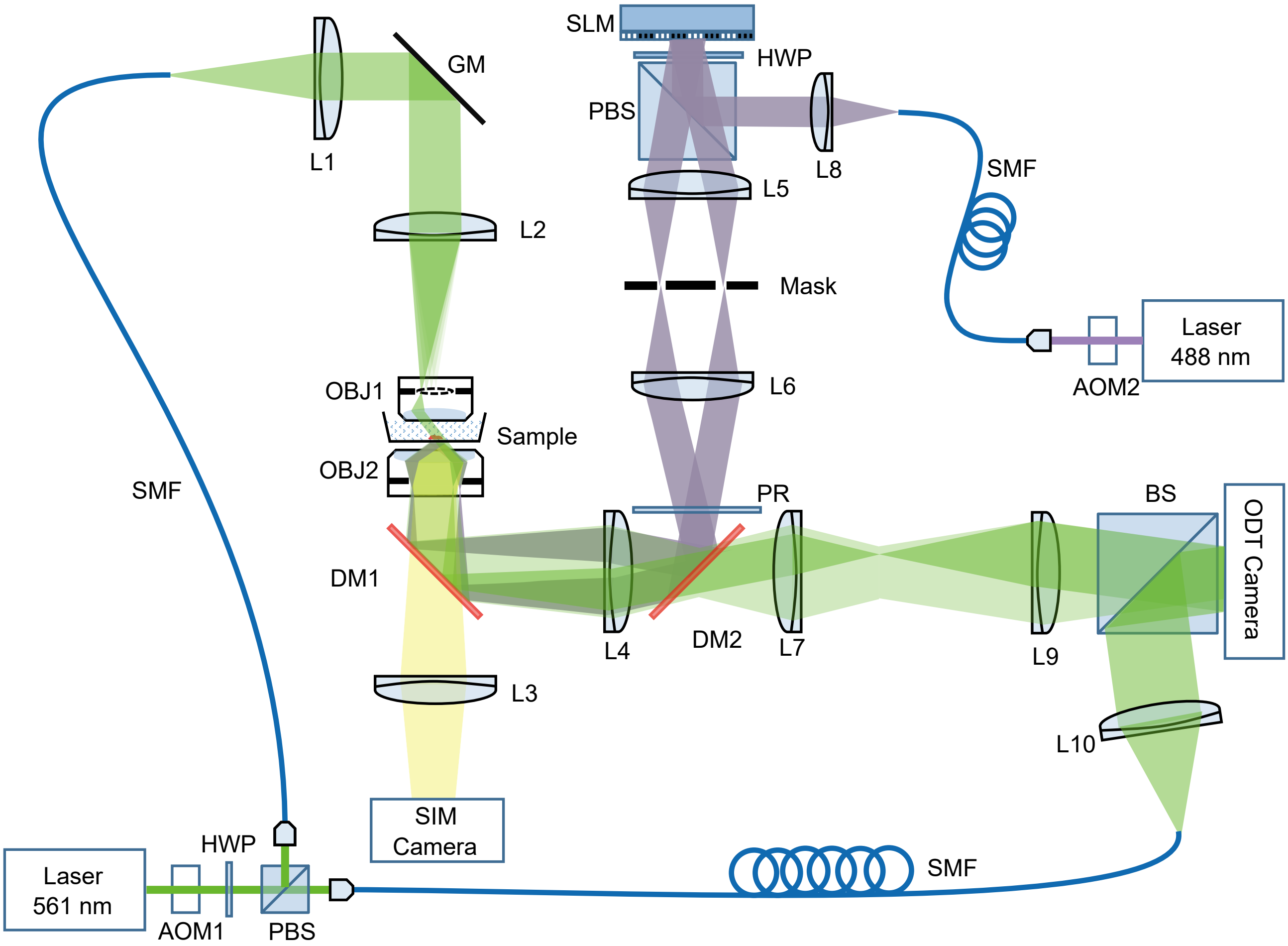

Laser 561: MSL-FN-561-50mW Changchun New Industries Optoelectronics Technology
Laser 488: Sapphire 488-200 CW Coherent
SMF: Single Mode Fiber
GM: (Galvo Mirror) GVS211/M Thorlabs
OBJ1: LUMPlanFLN 60x/1.0W Olympus
OBJ2: ApoN 100x/1.49Oil Olympus
Camera ODT: C13440 Hamamatsu OCRA-flash4.0 V3
Camera SIM: C11440 Hamamatsu OCRA-flash4.0 V2
SLM: QXDA-3DM Forth Dimension Displays
PR: (Half-Wave Variable Retarders) LCC1221-A Thorlabs
DM1: Dichroic Mirror ZT405/488/561/640-phase R, Chroma
DM2: Dichroic Mirror DMLP550L, Thorlabs
AOM1: AOM, CETC Chongqing Acousitc-Optic-Electric Co., LTD.
AOM2: AOTF, AA Opto-Electronic, France

L1: f = 40 mm
L2: f = 180 mm
L3: f = 180 mm
L4: f = 200 mm
L5: f = 500 mm
L6: f = 200 mm
L7: f = 180 mm
L8: f = 100 mm
L9: f = 250 mm
L10: f = 100 mm

Figure S2 | **Hardware configuration of SR-FACT microscope.** The green beam indicates the optic path of the ODT module, while the purple and yellow beams indicate the excitation and detection paths of the fluorescence Hessian SIM module.

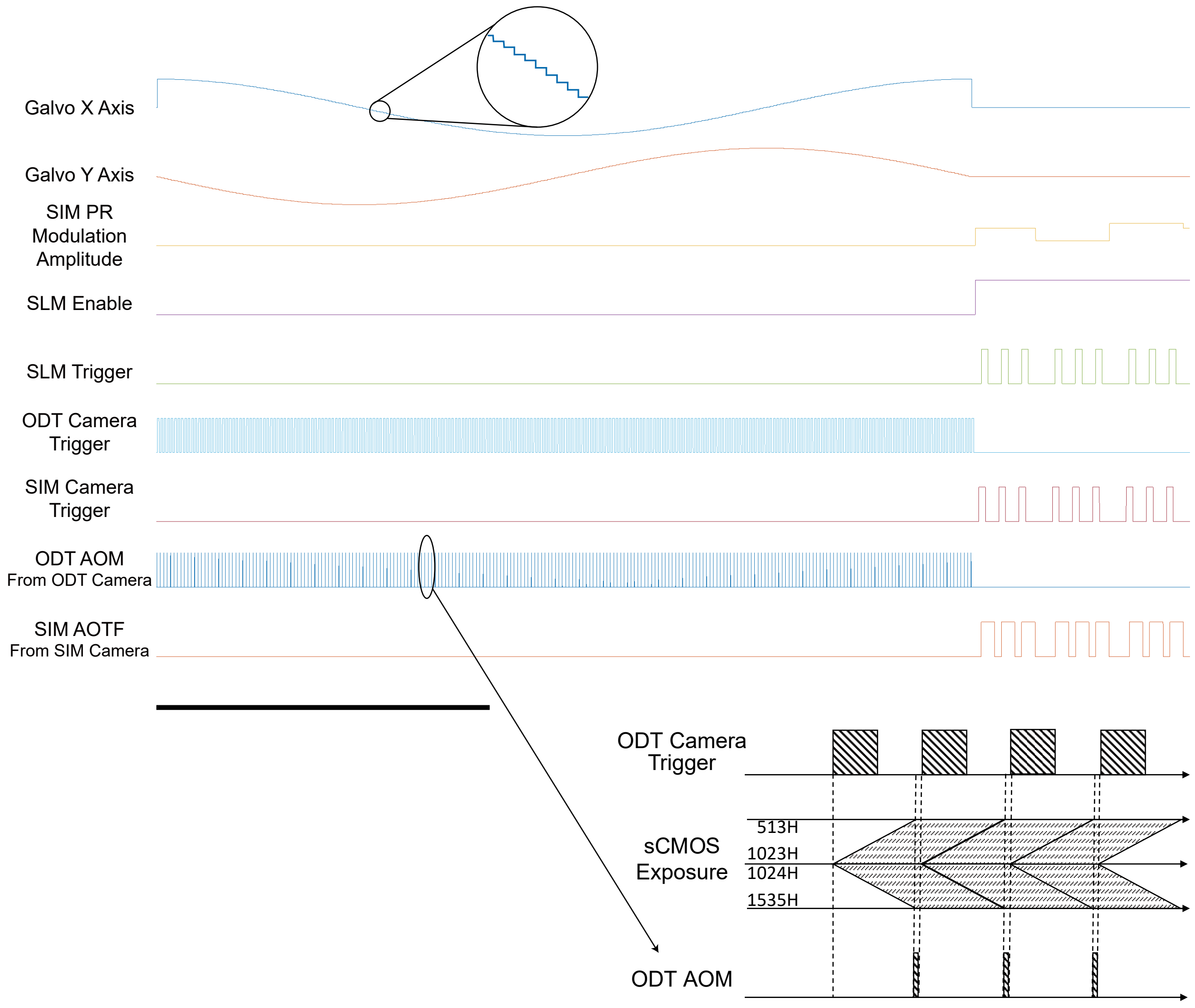

Figure S3 | Time sequence of synchronization for one cycle of ODT and SIM data acquisition. In one cycle, 240 holograms are recorded during the ODT imaging process, followed by 9 raw images taken to reconstruct one 2D-SIM image. Top inset: galvo-mirror driving signals to scan the beam during the acquisition of holograms at 240 illumination angles. Bottom inset: global exposure signals from ODT camera to switch on/off the AOM. Scale bar, 500 ms.

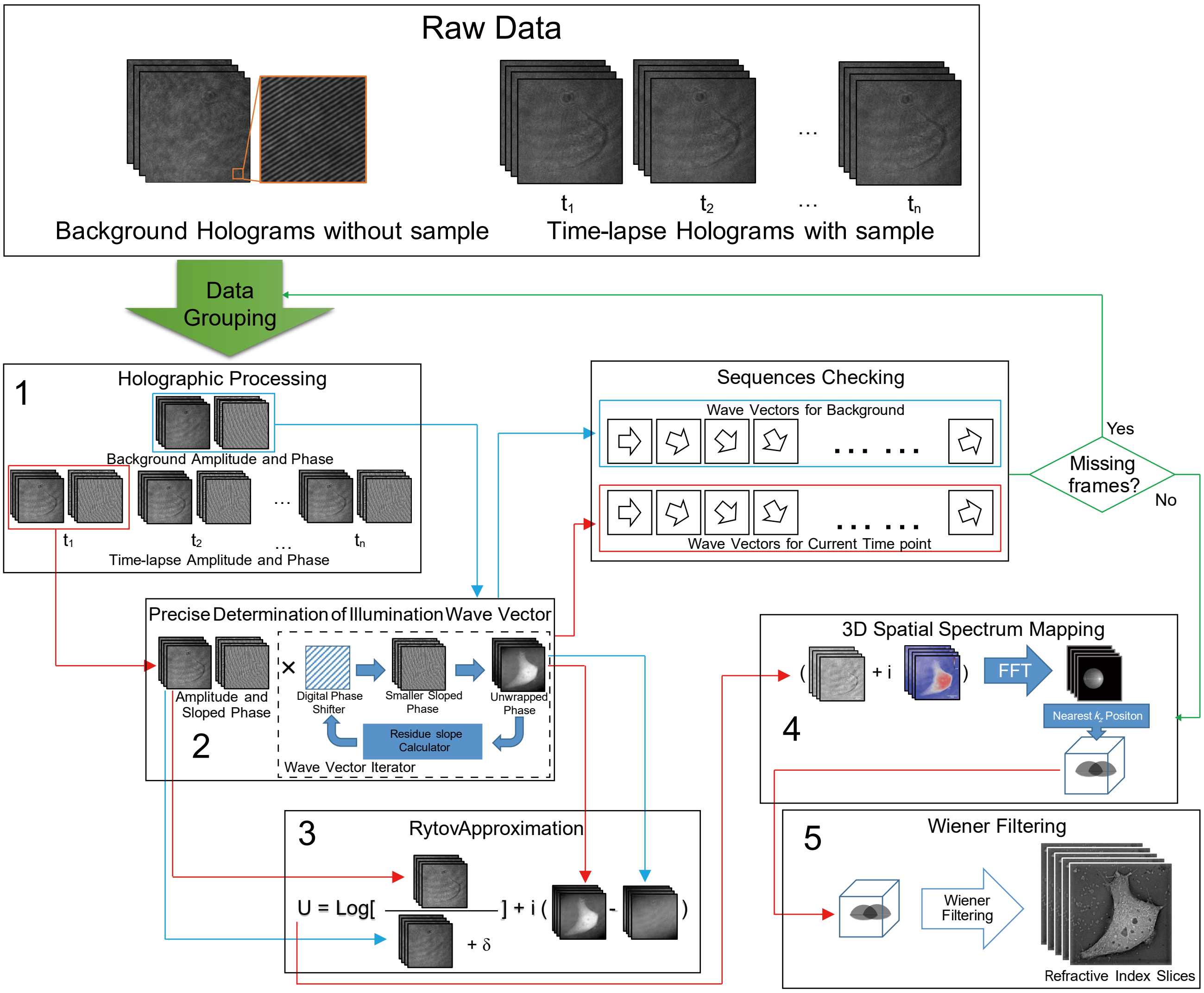

Figure S4 | **Pipeline of 3D reconstruction of ODT data.** We recorded one set of background ODT images in the absence of sample and another set in the presence of sample. The background data was sequentially processed as indicated by blue dashed lines, while red dashed lines highlight the processing flow. During data acquisition, there were missing frames occasionally during high speed data logging from camera. We used the pipeline indicated by green lines to detect and correct it offline.

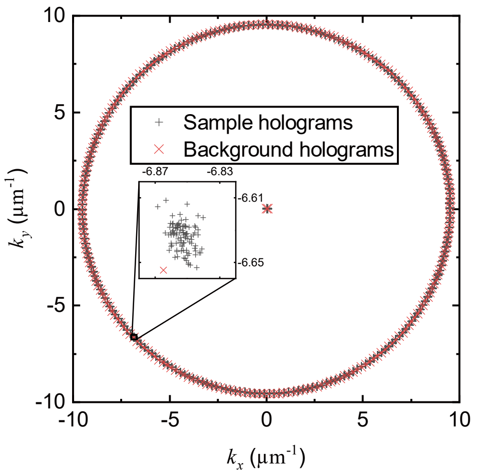

Figure S5 | Deviation of illumination wave vectors (k_x and k_y) at different time points. "×" marks wave vectors from the background holograms, while "+" marks wave vectors obtained during time-lapse imaging of a COS-7 cell at 0.8 Hz for 100 time points.

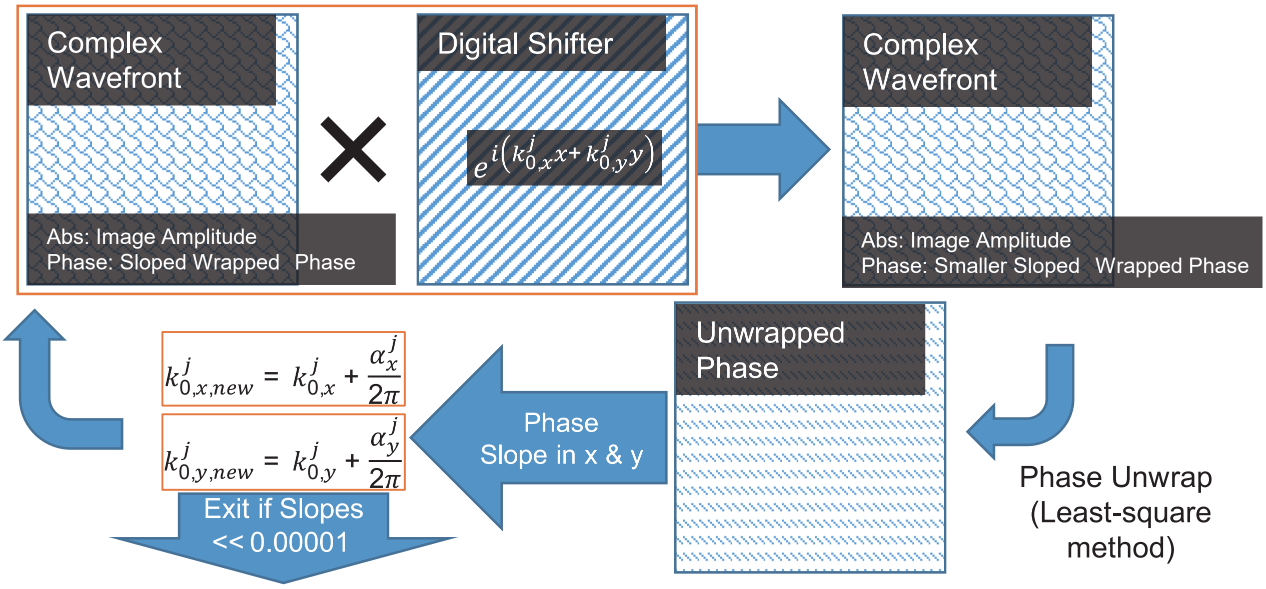

Figure S6 | Flow chart of VISA method for the precise determination of illumination wave vectors.

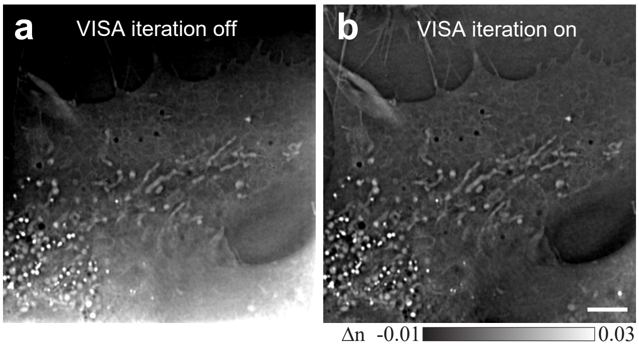

Figure S7 | ODT reconstructions with or without VISA processing. (**a-b**) Compared with reconstruction without VISA iteration (**a**), reconstruction of the live COS-7 cell with VISA processing is clearly more homogenous and superior (**b**). Scale bar, 5 µm.

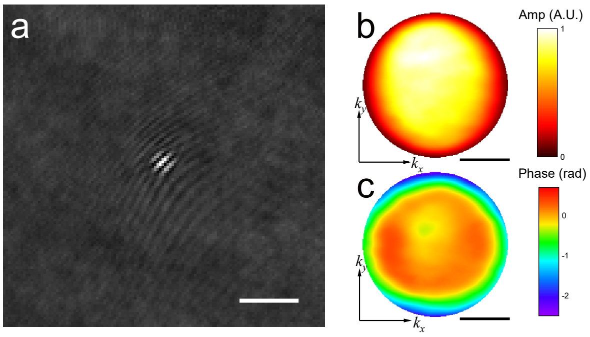

Figure S8 | Measured CTF of the microscope. (**a**) A raw holographic image of the artificial point source. (**b-c**) Calculated amplitudes (**b**) and phases (**c**) of 2D CTF from (**a**). Scale bars, (**a**) 1 μm and (**b-c**) $k = 2\pi / \lambda$.

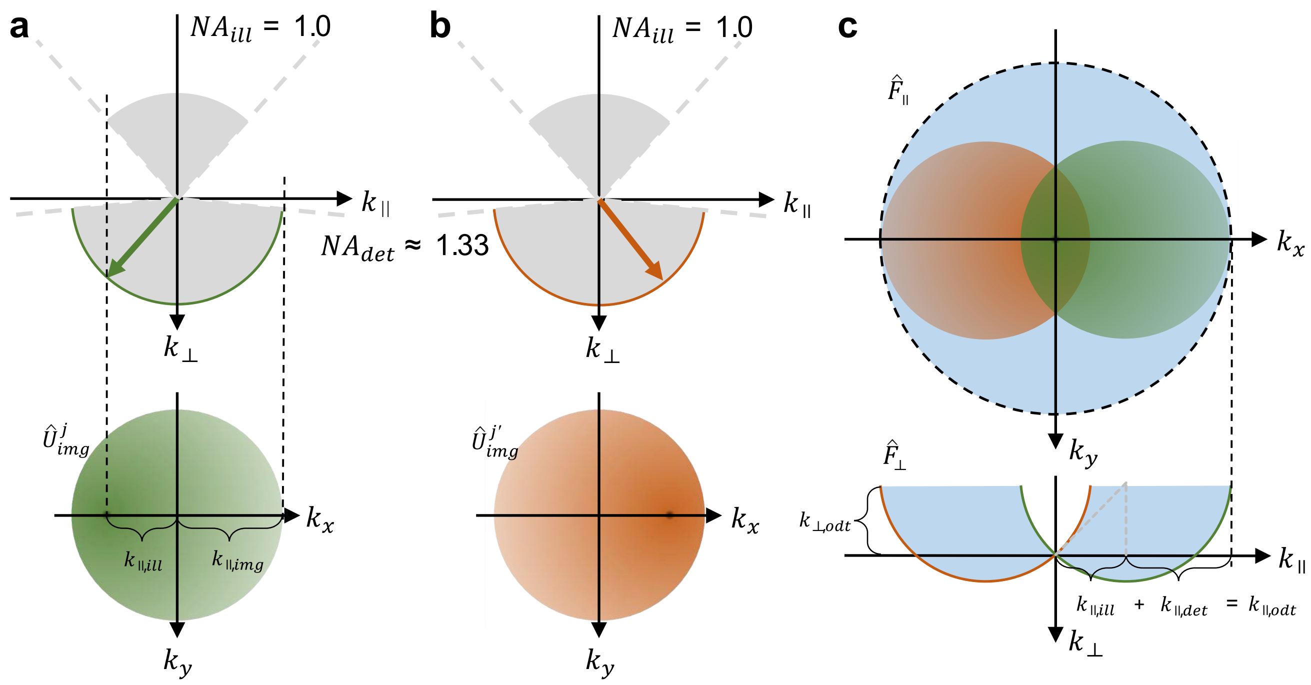

Figure S9 | Spatial frequency domain retrieved by ODT reconstruction. Two maximal illumination angles mirroring each other are selected as examples. Green and orange lines in the top diagrams in (**a**) and (**b**) indicate the illumination beams utilizing maximal NA of the illumination objective lens. Spatial frequency domain bandwidth is expanded, as shown in (**c**). The top panel in (**c**) shows the transverse spatial frequency bandwidth expansion, while the bottom panel in (**c**) shows nonuniform distribution of longitude frequency bandwidth. $k_{\parallel,ill}$ and $k_{\parallel,det}$ indicate transverse projections of maximal NA of illumination objective and detection objectives, respectively.

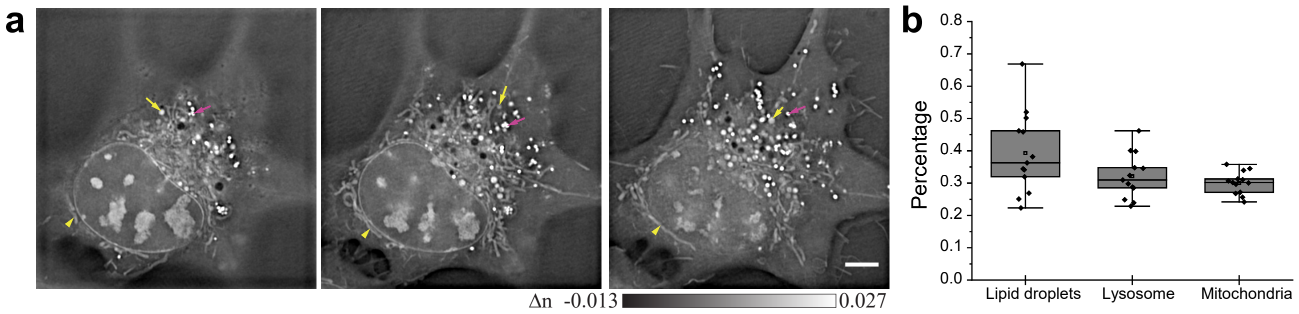

Figure S10 | Holistic view of organelles in 3D by ODT module. (**a**) Three different Z planes of a representative COS-7 cell. While yellow arrowheads indicate mitochondria, yellow and magenta arrows indicate LYs and LDs, respectively. (**b**) Average percentages of the area of LDs, LYs, and mitochondria within an axial volume of 0.86 μm (10 Z planes) to the total area of all LDs, LYs, and mitochondria within the cells (averages from 13 cells). Scale bar, 5 μm. Center lines, medians; limits, 75% and 25%; whiskers, maximum and minimum.

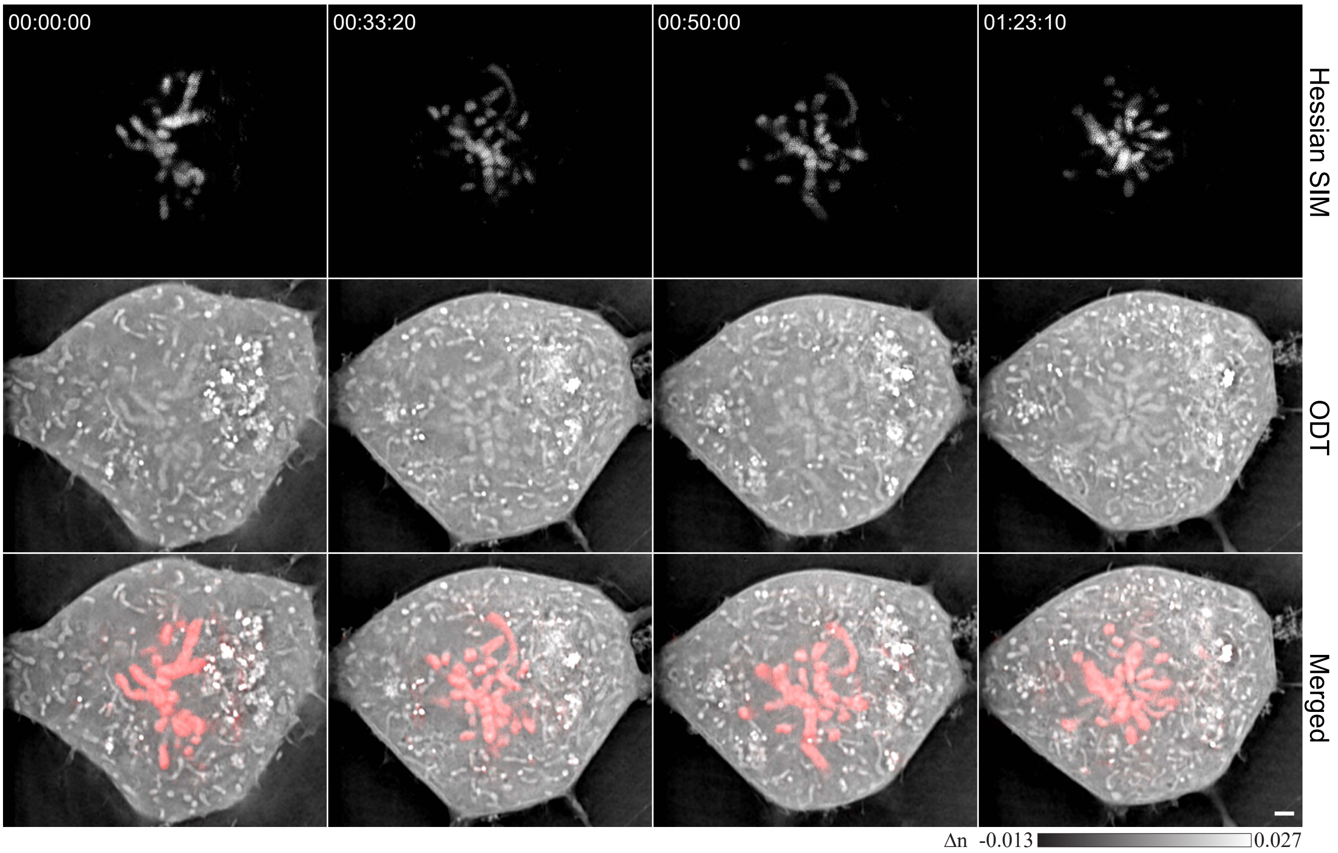

Figure S11 | A dividing COS-7 cell was arrested at late prophase after SR fluorescence imaging of chromosomes. The COS-7 cell was expressed with H2B-EGFP and arrested at late prophase for over an hour. This was in sharp contrast to the completed cell division observed in cells imaged only with ODT module (**Figure 2**). This phenomenon was observed 3 times in 3 different cells. Scale bar, 2 μm.

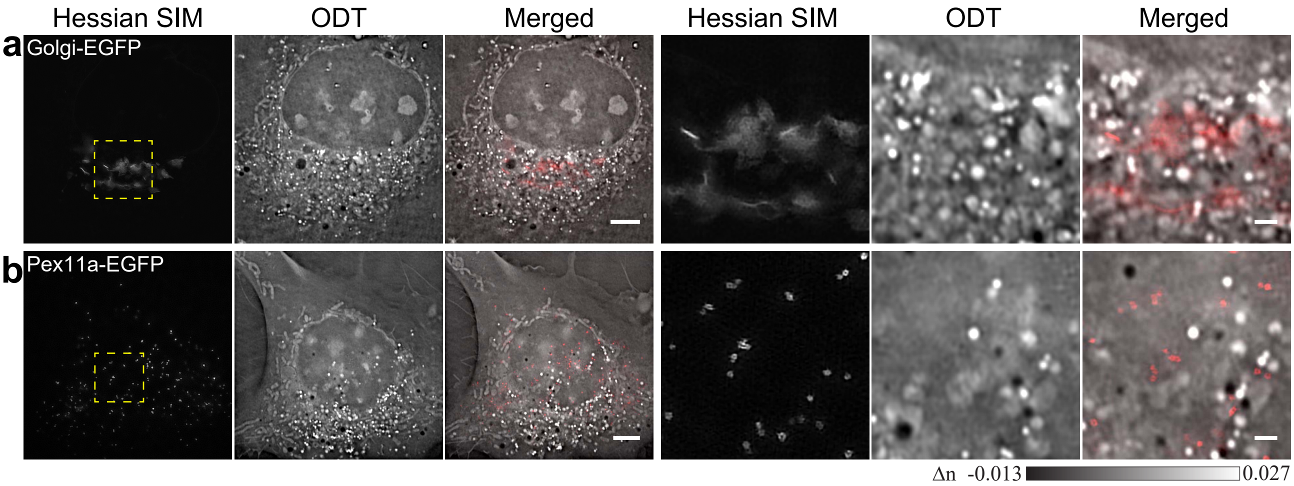

Figure S12 | Organelles not visible by ODT module. (**a**) Golgi in COS-7 cells labeled by β1,4-galactosyltransferase 1-EGFP failed to colocalize with any appreciable structures in ODT images. (**b**) Peroxisome in COS-7 cells labeled by Pex11-EGFP failed to colocalize with any appreciable structures in ODT images. Scale bars, 5 µm (left) and 1 µm (right).

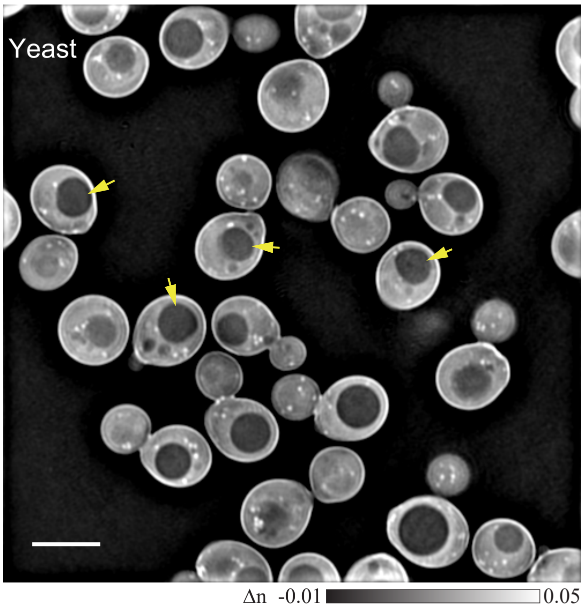

Figure S13 | Vacuoles in budding yeasts observed by ODT module. A few examples are highlight by yellow arrows. Scale bar, 5 µm.

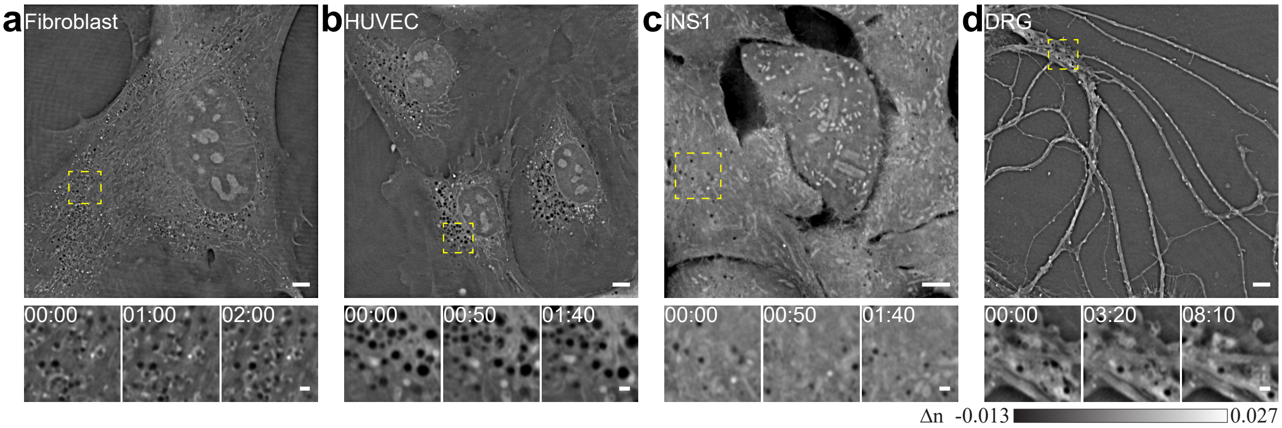

Figure S14 | DBs in different types of mammalian cells. (**a**) Human skin fibroblast cells. (**b**) Human umbilical vein endothelial cells. (**c**) Rat insulinoma INS-1 cells. (**d**) Mouse dorsal root ganglion neurons. Regions enclosed within yellow dashed boxes in the top panels were enlarged and shown in the bottom panels as montages, in which movement of DBs was obvious. All images shown here are representative of three batches of similar experiments. Scale bars, 5 μm (top) and 1 μm (bottom).

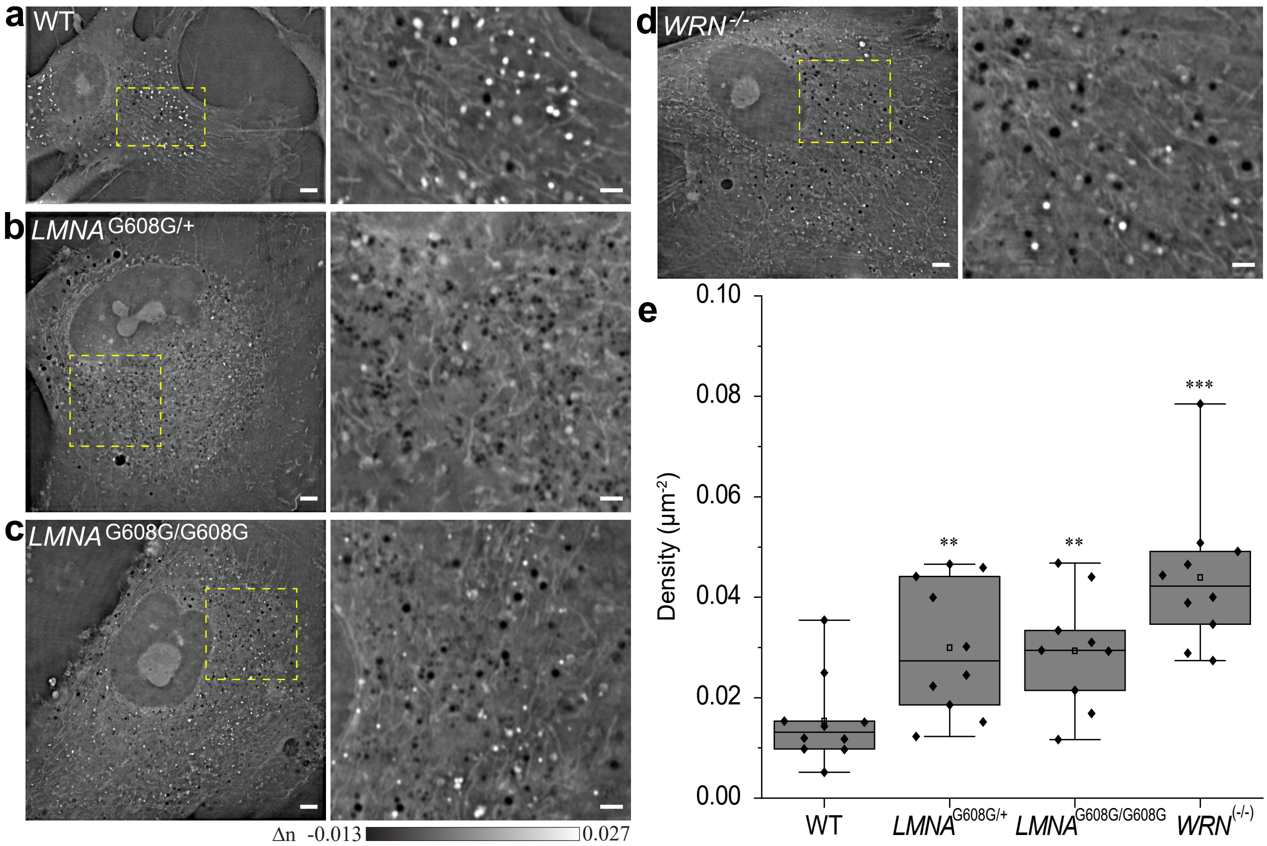

Figure S15 | Numbers of DBs in hMSCs are positively correlated with their aging phenotypes. We used WT-hMSCs (wild-type human MSCs as isogenic control), HGPS-hMSCs with heterozygous (*LMNA*$^{G608G/+}$) and homozygous (*LMNA*$^{G608G/G608G}$) *LMNA* mutation for modeling Hutchinson-Gilford progeria syndrome (HGPS) and WS-hMSCs with *WRN* deficiency (*WRN*$^{-/-}$) for modeling Werner syndrome (WS)[20, 21]. Representative ODT images of WT **(a)**, heterozygous (*LMNA*$^{G608G/+}$) **(b)**, homozygous (*LMNA*$^{G608G/G608G}$) **(c)**, and *WRN*$^{-/-}$ **(d)**. Corresponding DB densities at the center plane of cells are shown in **(e)**. Scale bar, 5 μm. Center lines, medians; limits, 75% and 25%; whiskers, maximum and minimum. We used the Mann-Whitney rank-sum test to analyze the data in **(e)**. * $p < 0.05$, ** $p < 0.01$, *** $p < 0.001$.

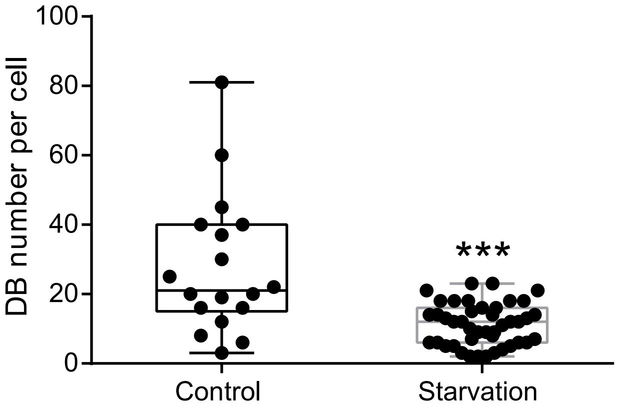

Figure S16 | Average number of DB in COS-7 cells decreased under starvation condition. For starvation experiments, COS-7 cells were incubated in complete medium or Hanks' balanced salt solution (HBSS, Gibco 14025) for 18 h prior to imaging.

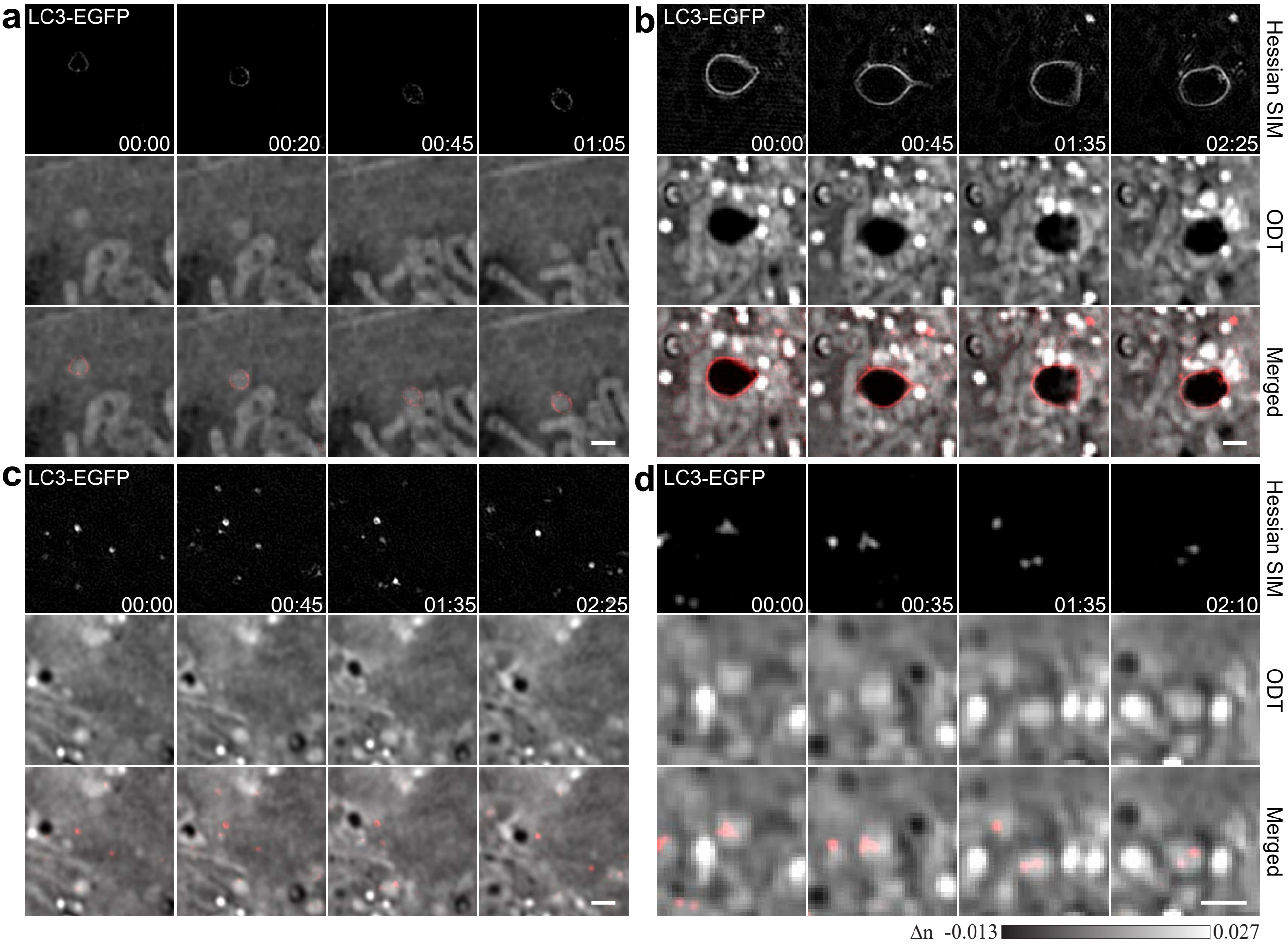

Figure S17 | Correlations between LC3-EGFP-labeled structures with DBs in COS-7 cells. (a-b) Some LC3-EGFP-labeled structures were fluorescent rings that either colocalize with the outer membrane of LYs (**a**) or large vacuolated vesicles (**b**). (**c-d**) Most of LC3-EGFP-labeled structures were fluorescent puncta, which did not overlapped with clear ODT structures (**c**), or overlapped with LE/LY structures (**d**). All images shown here are representative of three batches of similar experiments. Scale bars, 1 µm.

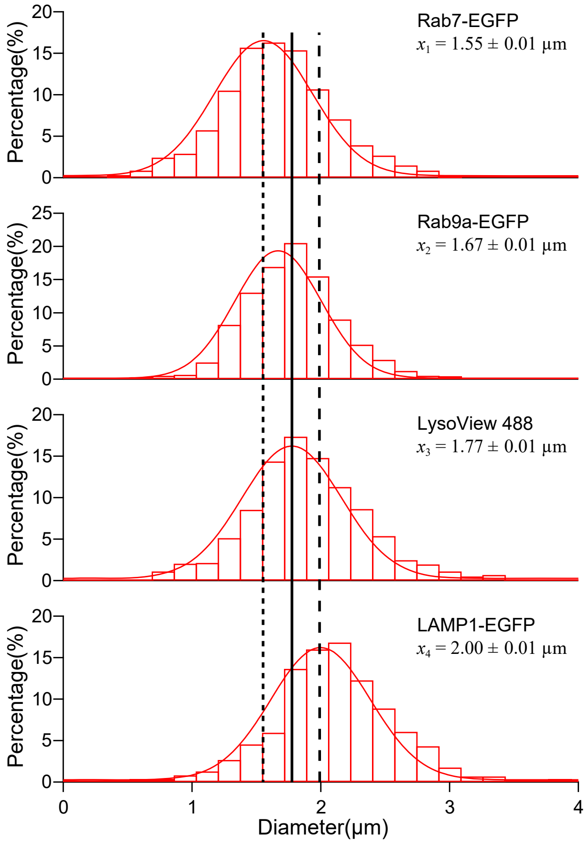

Figure S18 | Histograms of sizes of LE/LY structures observed by ODT module in COS-7 cells overexpressing different protein markers. All distributions could be fitted by the Gaussian function. In LysoView 488 labeled cells, the mean size of LE/LY structures was 1.77 ± 0.01 μm, which was smaller than that of cells overexpressing LAMP1-EGFP (2.00 ± 0.01 μm), and larger than those of cells overexpressing Rab7-EGFP (1.55 ± 0.01 μm) and Rab9a-EGFP (1.67 ± 0.01 μm).

Supplementary Table 1 | Imaging conditions used to obtain different figures.

	Label	ODT image time (s)	SIM image time (s)	Interval
**Figure 1d**	LifeAct-EGFP	1.22	0.27	3.51
**Figure 2**<br>**Supplementary Video S1-2**	NA	3	NA	7
**Figure 3a**<br>**Supplementary Video S3**	KDEL-EGFP	1.22	0.27	3.51
**Figure 3b**<br>**Supplementary Video S4**	MitoTracker Green			
**Figure 3c**<br>**Supplementary Video S5**	LipidSpot 488			
**Figure 3d**<br>**Supplementary Video S6**	LysoView 488			
**Figure 3e**<br>**Supplementary Video S7**	LaminA-EGFP			
**Figure 3f**<br>**Supplementary Video S8**	H2B-EGFP			
**Figure 4a**<br>**Supplementary Video S10**	NA	3	NA	2
**Figure 4b-c**<br>**Supplementary Video S11-12**	KDEL-EGFP	1.22	0.27	3.51
**Figure 4d-5g**<br>**Supplementary Video S13-16**	NA	1.22	NA	3.78
**Figure 5a-n**<br>**Supplementary Video S17-27**	NA	1.22	NA	3.78
**Figure 6a**	Rab5-EGFP	1.22	0.27	3.51
**Figure 6b**	EEA1-EGFP			
**Figure 6c**	FYVE-EGFP			
**Figure 6d**	Rab9a-EGFP			
**Figure 6e**	Rab7 -EGFP			
**Figure 6f**	LAMP1-EGFP			
**Figure 6g**	AQP2-EGFP			
**Figure S10**	NA	1.22	NA	3.78
**Figure S11**<br>**Supplementary Video S9**	H2B-EGFP	1.22	0.27	3.51
**Figure S12a**	β1,4-galactosyltransferase 1-EGFP	1.22	0.27	3.51
**Figure S12b**	Pex11-EGFP			
**Figure S13**	NA	1.22	NA	3.78
**Figure S14**	NA	3	NA	7

**Figure S15**<br>**Supplementary Video S28-31**	NA	3	NA	7
**Figure S16**	NA	3	NA	7
**Figure S17**	LC3-EGFP	1.22	0.27	3.51
